# Supplementary material for: Protocol for a 1-year prospective, longitudinal cohort study of patients undergoing Roux-en-Y gastric bypass and sleeve gastrectomy: the BARI-LIFESTYLE observational study
Source: BMJ Open. 2018 Mar 16;8(3):e020659. doi: 10.1136/bmjopen-2017-020659 (PMC5857659; doi:10.1136/bmjopen-2017-020659)
Supplement: Author's manuscript [file bmjopen-2017-020659.draft_revisions.pdf]

BMJ Open is committed to open peer review. As part of this commitment we make the peer review history of every article we publish publicly available.

When an article is published we post the peer reviewers' comments and the authors' responses online. We also post the versions of the paper that were used during peer review. These are the versions that the peer review comments apply to.

The versions of the paper that follow are the versions that were submitted during the peer review process. They are not the versions of record or the final published versions. They should not be cited or distributed as the published version of this manuscript.

BMJ Open is an open access journal and the full, final, typeset and author-corrected version of record of the manuscript is available on our site with no access controls, subscription charges or pay-per-view fees (http://bmjopen.bmj.com).

If you have any questions on BMJ Open's open peer review process please email info.bmjopen@bmj.com

BMJ Open

Protocol for a one-year prospective, longitudinal cohort study of patients undergoing Roux-en-Y gastric bypass and sleeve gastrectomy: the BARI-LIFESTYLE observational study.

Journal:	*BMJ Open*
Manuscript ID	bmjopen-2017-020659
Article Type:	Protocol
Date Submitted by the Author:	15-Nov-2017

Complete List of Authors:	Jassil, Friedrich; University College London, Centre for Obesity Research, Division of Medicine; University College London Hospitals, Bariatric Centre for Weight Management and Metabolic Surgery<br>Carnemolla, Alisia; University College London, Centre for Obesity Research, Division of Medicine; National Institute of Health Research, University College London Hospitals Biomedical Research Centre<br>Kingett, Helen; University College London Hospitals, Bariatric Centre for Weight Management and Metabolic Surgery; National Institute of Health Research, University College London Hospitals Biomedical Research Centre<br>Paton, Bruce; Institute of Sport, Exercise and Health<br>O'Keeffe, Aidan; University College London, Department of Statistical Science<br>Doyle, Jacqueline; University College London Hospitals, Bariatric Centre for Weight Management and Metabolic Surgery; National Institute of Health Research, University College London Hospitals Biomedical Research Centre<br>Morris, Stephen; University College London, Department of Applied Health Research<br>Lewis, Neville; University College London, The Hatter Cardiovascular Institute, Institute of Cardiovascular Science<br>Kirk, Amy; University College London Hospitals, Bariatric Centre for Weight Management and Metabolic Surgery; National Institute of Health Research, University College London Hospitals Biomedical Research Centre<br>Pucci, Andrea; University College London Hospitals, Bariatric Centre for Weight Management and Metabolic Surgery; University College London, Centre for Obesity Research, Division of Medicine<br>Chaiyasoot, Kusuma; University College London, Centre for Obesity Research, Division of Medicine; University College London Hospitals, Bariatric Centre for Weight Management and Metabolic Surgery<br>Batterham, Rachel; University College London, Centre for Obesity Research, Division of Medicine; University College London Hospitals, Bariatric Centre for Weight Management and Metabolic Surgery

Keywords:	Obesity, Bariatric Surgery, Body Composition, Physical Activity, Diet, Quality of Life

For peer review only

Protocol for a one-year prospective, longitudinal cohort study of patients undergoing Roux-en-Y gastric bypass and sleeve gastrectomy: the BARI-LIFESTYLE observational study.

Friedrich C. Jassil [1,2], Alisia Carnemolla [1,3], Helen Kingett [2,3], Bruce Paton [4], Aidan G. O'Keeffe [5], Jaqueline Doyle [2,3], Stephen Morris [6], Neville Lewis [7], Amy Kirk [2,3], Andrea Pucci [1,2], Kusuma Chaiyasoot [1,2] and Rachel L. Batterham [1,2,3*]

Author affiliations

[1] Centre for Obesity Research, Division of Medicine, University College London, London, United Kingdom

[2] University College London Hospitals, Bariatric Centre for Weight Management and Metabolic Surgery, London, United Kingdom

[3] National Institute of Health Research University College London Hospitals Biomedical Research Centre, London, United Kingdom

[4] Institute of Sport, Exercise and Health, London, United Kingdom

[5] Department of Statistical Science, University College London, London, United Kingdom

[6] Department of Applied Health Research, University College London, London, United Kingdom

[7] The Hatter Cardiovascular Institute, Institute of Cardiovascular Science, University College London, London, United Kingdom

*Corresponding author: Professor Rachel L. Batterham; r.batterham@ucl.ac.uk

Address: Centre for Obesity Research
Division of Medicine
University College London
Rayne Building, 5 University Street
London WC1E 6JF, United Kingdom

For peer review only
For peer review only - http://bmjopen.bmj.com/site/about/guidelines.xhtml

ABSTRACT

Introduction: Roux-en-Y gastric bypass and sleeve gastrectomy are the two most common bariatric surgery performed in the UK that result in comparable weight loss and remission of obesity-associated co-morbidities. However, there is a paucity of studies examining the impact of these procedures upon body composition, physical activity levels, sedentary behaviour, physical function and strength, dietary intake, health-related quality of life and costs.

Methods and analysis: The BARI-LIFESTYLE observational study is a one-year prospective, longitudinal cohort study aiming to recruit 100 patients with severe obesity undergoing either primary Roux-en-Y gastric bypass or primary sleeve gastrectomy from two bariatric centres in London, UK. Participants will be followed-up four times during the study period; pre-surgery baseline (T0) and at 3 (T1), 6 (T2), and 12-month (T3) post-surgery. In addition to the standard follow-up investigations, assessments including dual-energy X-ray absorptiometry scan, bioelectric impedance analysis, 6-minute walk test, sit-to-stand test, and handgrip test will be undertaken together with completion of questionnaires. Physical activity levels and sedentary behaviour will be assessed using accelerometer, and dietary intake will be recorded using a 3-day food diary. Outcome measures will include: body weight, body fat mass, lean muscle mass, bone mineral density, physical activity levels, sedentary behaviour, physical function and strength, dietary intake, health-related quality of life, remission of co-morbidities, healthcare resource utilisation and costs.

Ethics and dissemination: This study has been reviewed and given a favourable ethical opinion by London-Dulwich Research Ethics Committee (17/LO/0950). The results will be presented to stakeholder groups locally, nationally and internationally and published in peer-reviewed medical journals. The lay-person summary of the findings will be published on the Centre for Obesity Research, University College London website (http://www.ucl.ac.uk/obesity).

For peer review only

For peer review only - http://bmjopen.bmj.com/site/about/guidelines.xhtml

Strengths and limitations of this study

- A comprehensive prospective, longitudinal study with detailed assessments undertaken prior to and for one year following bariatric surgery examining changes in body composition, physical activity (PA) levels, sedentary behaviour, physical function and strength, dietary intake, health-related quality of life and costs, relative to baseline pre-surgery.
- The use of validated research tools (accelerometer to assess PA levels and sedentary behaviour, dual-energy X-ray absorptiometry [DXA] scan to assess body composition and validated questionnaires) will generate high-quality data.
- A potential sample selection bias due to exclusion of patients with functional limitation and/or non-ambulatory and patients with more than 200 kg of body weight owing to the weight limit of the DXA scan.
- A relatively small sample size, nevertheless, this number is adequate to generate in-depth insights into the various outcomes of Roux-en-Y gastric bypass and sleeve gastrectomy as delivered in the UK healthcare setting.

For peer review only - http://bmjopen.bmj.com/site/about/guidelines.xhtml

INTRODUCTION

Bariatric surgery engenders marked sustained weight loss and is recommended by the National Institute for Health and Care Excellence (NICE) as a treatment option for people of severe obesity[1], estimated to affect approximately 2.6 million adults in the UK[2]. Roux-en-Y gastric bypass (RYGB) and sleeve gastrectomy (SG) are now the two most common procedures performed in the UK, which result in comparable weight loss and remission of obesity associated co-morbidities[3]. However, there is a paucity of studies examining the impact of these procedures upon body composition, particularly bone mineral density (BMD), physical activity (PA) levels, sedentary behaviour, physical function and strength, dietary intake, health-related quality of life (HRQoL) and costs.

Bariatric surgery leads to a marked decrease in fat mass (FM), but fat free mass (FFM) particularly bone mass is also reduced post-surgery[4], potentially negatively impacting on physical function and strength, and putting patients at increased risk of osteoporotic fracture in the future[5 6]. Surgical modification of the gastrointestinal tract impairs the intake and/or absorption of essential nutrients for bone health that consequently perturbs bone metabolism, leading to BMD deterioration[5-8]. Significant bone mass loss has been reported to occur rapidly in the first year of surgery and continues to deteriorate up to 3 years even after maximum weight loss has been achieved[7]. However, these data, are mainly based on studies undertaken in patients who underwent RYGB whereas SG is now the most common procedure undertaken both in the UK and globally[3 9]. Currently, it is unclear whether the rate of bone mass loss following SG paralells weight loss[10-12]. Given that the number of younger patients and women of childbearing age undergoing bariatric surgery continues to increase and BMD measurement is not a routine follow-up investigation[13], there is an urgent need to assess the impact of RYBG and SG on bone health in the UK bariatric population.

For peer review only - http://bmjopen.bmj.com/site/about/guidelines.xhtml

Adherence to a post-bariatric lifestyle changes is the cornerstone of a successful weight loss[14]. Studies have shown that greater PA, lower sedentary time and high compliance to dietary recommendation post-surgery associate with greater weight loss, preservation of lean muscle mass (LMM) and bone mass, as well as improvement in HRQoL[15-18]. However, patients spend 80% of their waking time in sedentary behaviour post-surgery[19], activity that associates with increased risk of cardiometabolic disease and mortality[20]. Following surgery, patients are advised to undertake at least 150 minutes of moderate-to-vigorous physical activity (MVPA) per week, a duration and intensity that are recommended to reap the metabolic benefit of PA[21]. However, objectively measured MVPA decreases post-surgery with only 10% of patients achieving the recommended MVPA levels[22]. Likewise, a recent study undertaken in the UK has reported that weight loss post-surgery did not correspond to improvement in MVPA and sedentary behaviour. However, the small sample size of this study (n=22) together with relatively short follow-up period limited its generalisability[23]. Further studies are therefore required to expand the information in this regard. In terms of dietary recommendations, daily protein intake of 60 g or more post-surgery is crucial for increasing satiety, preserving LMM, improving body composition and preventing against weight regain[24-27]. However, most patients are unable to achieve this in the first postoperative year, the period when rapid weight loss occurs[28]. Whether this is also the case for UK bariatric population is not known as no such data has ever been reported thus far[28].

For peer review only

Impaired HRQoL is common in obesity[29] and often one of the driving factors for seeking weight loss surgery[30]. HRQoL is defined as individuals' perception of well-being that refers to physical, psychological and social domains of health[31]. Most studies reported improvement in all HRQoL domains with greater scores observed in the first post-operative year although some studies showed that the improvement is limited to only the physical domain but not the mental health component of HRQoL[32]. Despite mounting evidence in the international literature reporting the beneficial impact of bariatric surgery on HRQoL, data from the UK bariatric population does not exist[33]. There is some

For peer review only - http://bmjopen.bmj.com/site/about/guidelines.xhtml

evidence that bariatric surgery can reduce in cost savings that offset the initial costs of surgery, though little UK evidence for RYGB and SG[34-36].

Taken together the lack of post-operative data coupled with recommendations from systematic reviews[22 28] provide a strong rational to undertake a prospective study to evaluate the impact of RYGB and SG upon body composition particularly BMD, PA levels, sedentary behaviour, physical function and strength, dietary intake, HRQoL and costs in a UK bariatric population. Information gained from this study will provide valuable data to inform the implementation of future post-surgery lifestyle programmes with the aim of maximising the beneficial outcomes of bariatric surgery as highlighted by NICE[1]. This paper details the study design and outcomes of the BARI-LIFESTYLE observational study.

OBJECTIVES

The overall objective of BARI-LIFESTYLE observational study is to evaluate the impact of RYGB and SG on changes in body weight, body composition, PA levels, sedentary behaviour, physical function and strength, dietary intake, HRQoL, remission of co-morbidities, healthcare resource utilisation and costs in a cohort of 100 patients.

The specific objectives are to evaluate post-surgery changes in:

1. Percentage weight loss (%WL) at one year post-surgery, relative to baseline pre-surgery weight.
2. Body fat mass, assessed using dual-energy X-ray absorptiometry (DXA) scan and bioelectrical impedance analysis (BIA), relative to pre-surgery at 12 months post-surgery.
3. LMM, assessed using DXA scan and BIA, relative to pre-surgery at 12 months post-surgery.
4. BMD, assessed using DXA scan and BIA, relative to pre-surgery at 12 months post-surgery.

For peer review only - http://bmjopen.bmj.com/site/about/guidelines.xhtml

5. PA levels (light, moderate, vigorous), percentage achieving 150 minutes of MVPA in a week and sedentary behaviour assessed using accelerometer at 3, 6, and 12 months post-surgery, relative to pre-surgery.
6. Physical function and strength assessed using 6-minute walk test (6MWT), sit-to-stand (STS) test and handgrip test at 3, 6 and 12 months post-surgery, relative to pre-surgery.
7. Dietary intake assessed using food diary at 3, 6 and 12 months post-surgery, relative to pre-surgery.
8. HRQoL assessed using EuroQol-5D-3L (EQ-5D-3L) and Impact of Weight on Quality of Life-Lite (IWQOL-Lite) at 3, 6 and 12 months post-surgery, relative to pre-surgery.
9. Characteristics of attitude and symptoms of depression assessed using Beck Depression Inventory-II (BDI-II) at 3, 6 and 12 months post-surgery, relative to pre-surgery.
10. Obesity-associated comorbidities (type 2 diabetes [T2D], dyslipidaemia, hypertension, obstructive sleep apnoea [OSA]) at 3, 6 and 12 months post-surgery, relative to pre-surgery.
11. Healthcare resource utilisation and costs assessed using an adapted version of the Client Service Receipt Inventory (CSRI) at 3, 6 and 12 months post-surgery, relative to pre-surgery.

METHODS AND ANALYSIS

Study design and setting

BARI-LIFESTYLE observational study is a prospective, longitudinal cohort study of patients undergoing bariatric surgery conducted in London, UK (Figure 1). A total of 100 patients will be recruited over a 2-year period from 2018 to 2019, and will be followed for up to 12 months post-surgery. Recruitment will take place at the Bariatric Centre for Weight Management and Metabolic Surgery, University College London Hospitals (UCLH) (study site) and the Bariatric and Obesity Surgery Clinic at the Whittington Hospital that acts as a participant identification centre (PIC). Participants recruited from the Whittington Hospital will have their surgical procedure undertaken at the same centre, but all study procedures such as written informed consent, baseline assessment,

For peer review only - http://bmjopen.bmj.com/site/about/guidelines.xhtml

post-surgery follow-up care and study assessments will be undertaken by the bariatric team at UCLH. This study is carried out by the Centre for Obesity Research, Division of Medicine, University College London (UCL), with an expected total duration of 36 months, from the first participant enrolled to last participant follow-up.

Participants and recruitment

Patients who are planned to undergo either primary RYGB or primary SG will be screened for suitability for the study by the bariatric team at the study site and PIC based on the inclusion and exclusion criteria when they attend the standard pre-surgical assessment (Table 1). Verbal consent will be sought from those fulfilling the eligibility criteria and interested in participating to be approached by a research investigator. Patients will be given a participant information sheet inviting them to participate in a one-year prospective, longitudinal cohort study looking at the effect of bariatric surgery on body weight, body composition, PA levels, sedentary behaviour, physical function and strength, dietary intake, HRQoL, remission of co-morbidities, healthcare resource utilisation and costs. Consented participants will then be scheduled to attend a baseline assessment, approximately 6 weeks before surgery day at the study site. Each participant will be given a Fitbit Alta HR to enable them to self-monitor their activity levels and to reduce sedentary behaviour. Based on the weekly number of bariatric procedures undertaken at UCLH and Whittington Hospital and after considering the eligibility criteria, estimated recruitment rate is approximately 7 participants per month. Hence, the expected recruitment period for the study is 15 to 20 months.

All participants will receive the standardised post-bariatric care as stipulated by NICE[1]. Participants will attend the study site for monitoring of nutritional intake, vitamin and mineral deficiencies, comorbidities and medication review. Participants will receive verbal PA and dietary advice from a specialist nurse and dietitian at weeks 12 and 36 post-surgery, respectively.

For peer review only - http://bmjopen.bmj.com/site/about/guidelines.xhtml

Outcomes measures

Outcome measures will be collected at four study time points, designed to coincide with normal follow-up care visits; baseline visit at approximately 6 weeks before surgery (T0) then at 3 (T1), 6 (T2), and 12-month (T3) post-surgery (Table 2).

Sociodemographic, medical history, and physical examination

Participants' sociodemographic data, medical history, and physical examination will be completed by the bariatric team at the baseline visit. Data to be captured including age, gender, ethnicity, educational level, marital status, medication intake, weight history, pregnancy history, alcohol consumption using the AUDIT-C questionnaire[37], smoking habits and family history of obesity and comorbidities.

Primary outcome

Body weight will be measured using a weighing scale (Model VT200/220, Vishay Transducers Ltd., CA, USA) with participants wearing light clothes and without shoes and heavy accessories, to the nearest 0.1 kg. Similarly, height will be determined using a stadiometer (Seca 217, Seca GmbH & Co. KG, Hamburg, Germany) to the nearest 0.01 m. %WL will be calculated using the following formula: %WL = [(weight on the day of surgery – weight at time-point after surgery)/weight on the day of surgery] × 100, measured at each study time point.

Secondary outcomes

Body composition (body fat mass, LMM and BMD)

Body composition will be assessed at baseline and 12-months post-surgery using DXA scan (Horizon W DXA system, Hologic, Inc., MA, USA). DXA scan uses ionising radiation to measure different body compartments. This is the current reference standard for assessing body composition and a gold standard method to diagnose osteopenia and osteoporosis[38]. In addition, body composition will be

For peer review only - http://bmjopen.bmj.com/site/about/guidelines.xhtml

measured using BIA (Tanita DC-430MAS, Tanita Corp., Tokyo, Japan) at each study visit. This is a non-invasive, easy to perform and cheaper option to measure body composition that is based on the differences in electrical conductivity of FM and FFM tissues[39].

PA levels and sedentary behaviour

PA and time spent in light, moderate and vigorous activities, and sedentary behaviour will be measured objectively using ActiGraph wGT3X-BT (Pensacola, FL, USA), an accelerometer-based activity monitor[40]. Participants will be instructed to wear the ActiGraph on their dominant hip for one week, from waking in the morning until going to bed at night, and to remove it only during water-based activities. Additionally, participants will be asked to keep an activity diary throughout the week, to assist interpretation of data from the device. Both the device and activity diary have to be returned to the investigators for data analysis (ActiLife 6 software, Pensacola, FL, USA).

Physical function and strength

Participants' functional capacity will be assessed using a 6MWT, a self-paced, submaximal assessment of functional capacity used to prescribe appropriate exercise[41]. Lower body functional capacity and strength will be assessed using the STS test[42]. Static muscle strength will be assessed using Jamar Hydraulic Hand Dynamometer (Petterson Medical, IL, USA)[43].

Dietary intake

All participants will be required to keep a 3-day food diary (2 working days and 1 weekend day) for one week at each study time point. This method has a higher agreement with the 9 days food dairy compared to the food frequency questionnaire[44] whilst reducing the burden to patients and thus promoting better compliance for documenting food intake. The completed food diary will be returned to the investigators together with the ActiGraph and activity diary by using a stamped addressed envelope provided to participants.

For peer review only - http://bmjopen.bmj.com/site/about/guidelines.xhtml

HRQoL

HRQoL will be assessed using EQ-5D-3L and IWQOL-Lite. The EQ-5D-3L descriptive system is a 5-item self-report questionnaire that assesses the following domains: mobility, self-care, usual activities, pain/discomfort and anxiety/depression, and a visual analogue scale, which records self-rated health on a 0 to 100 scale[45]. EQ-5D-3L health states will be converted into utility values using a formula that attaches weights to each level in each dimension based on valuations by general population samples. We will use a value set for the UK population to calculate utility values at each time point for every participant[46]. The IWQOL-Lite is a 31-item, self-report, obesity and overweight-specific measure of HRQoL[47]. This tool consists of a total score and scores on each of five scales – physical function, self-esteem, sexual life, public distress, and work; higher scores indicate better HRQoL.

Attitude and symptoms of depression

BDI-II is a 21-item self-report questionnaire that assesses mood over the past week[48]. Symptoms of depression are classified by the total score: minimal, mild, moderate, and severe symptoms.

Obesity-associated comorbidities

Co-morbidities (T2D, dyslipidaemia, hypertension, OSA) and medication review will be carried out at each study time point.

Healthcare resource utilisation and costs

Resource use data will be collected using an adapted version of the CSRI[49], including the costs of bariatric surgery plus pre-surgery visits, number of contacts with healthcare professionals, visits to specialist clinics, the emergency department, admissions to the hospital, primary care contacts, and medications. Resource use data will be converted into costs using published unit costs[50-52]. In addition, information regarding support from informal carers, employment status and time off work

For peer review only - http://bmjopen.bmj.com/site/about/guidelines.xhtml
For peer review only

will be collected. Resource use data will be collected for the previous 6 months at the baseline visit and since participants' last study visit at each post-surgery study time point.

Statistical analysis

The demographic and medical history information collected at baseline shall be presented in a table. Categorical variables shall be reported as raw numbers and percentages. Reports of continuous variables shall include mean, median, range and standard deviation.

Primary outcome analysis

The primary outcome is the %WL measured longitudinally at baseline and 12 months post-surgery. %WL will be analysed using a linear mixed effects model over the three post-surgery time points (3 months, 6 months and 12 months) after controlling for the baseline body weight measure and height. Model assumptions shall be checked and suitable transforms of the primary outcome variable considered if necessary. In addition, overall percentage change in weight since baseline shall be computed marginally at each of 3, 6 and 12 months and displayed graphically.

Secondary outcomes analyses

Analyses of longitudinal secondary outcomes shall be performed using linear mixed effects regression models, with a normal distribution assumed for continuous outcomes (or a suitable transform of these outcomes). Model parameter estimates together with appropriate 95% confidence intervals shall be reported. Categorical outcomes (e.g. proportions of participants with co-morbidities) shall be summarised in tabular form at each time point. Where appropriate (for example, for proportions), estimates and 95% confidence intervals will be presented. To analyse costs, we will assume the costs measured at baseline for the preceding 6 months would persist during follow-up in the absence of surgery; we will then compare post-surgery costs with predicted

For peer review only - http://bmjopen.bmj.com/site/about/guidelines.xhtml

costs that would have been incurred in the absence of surgery. To account for skewness of the cost data, we will use a generalised linear model with gamma family and log link[53].

Missing data

Bias due to missing data will be investigated by comparing the baseline characteristics of participants with and without missing values. Depending on the extent of missingness, the predictors of missing values will be identified. The primary outcome analysis will be adjusted for those predictors of missing values, which are related to missingness. Multiple imputation using chained equations shall be considered as part of a sensitivity analysis for missing data in the primary outcome model.

Data storage and retention

All data will be handled in accordance with the UK Data Protection Act 1998. Physical data will be stored in a secure room with limited access to only members of the research team, whereas computers storing electronic data will be encrypted and password protected. Each participant will be given a unique study identification number and used on their records instead of their name. The master list linking participants' name and the study identification number will be kept in a secure location. This way, participants' personal identity and data collected in the study cannot be linked by anyone outside the study team. This study is registered with the UCL Data Protection (Reference: Z6364106/2017/04/43). At the end of the study, all essential documentation will be archived securely for a minimum of 20 years from the declaration of the end of study.

ETHICS AND DISSEMINATION

This study has been reviewed and given a favourable ethical opinion by London-Dulwich Research Ethics Committee (Reference: 17/LO/0950). Potential participants will be explained in detail regarding the aims, methods, anticipated advantages and disadvantages of participation in the study by Good Clinical Practice (GCP) trained investigators prior to obtaining their written informed

For peer review only - http://bmjopen.bmj.com/site/about/guidelines.xhtml

consent. Participants will be informed that their participation is on a voluntary basis, and they have the right to withdraw from the study at any time without affecting their present and future medical care. No research procedures will be undertaken prior to patients giving written informed consent. As a duty of care, all possible adverse events will be collected from the day participants consented for the study to monitor their safety.

The findings will be presented to stakeholder groups locally, nationally and internationally and published in peer-reviewed medical journals. The lay-person summary of the findings will be published on the Centre for Obesity Research, University College London website (http://www.ucl.ac.uk/obesity). The results will be fully anonymised, and none of the participants will be identified in any report or publication.

For peer review only

ADVANTAGE AND LIMITATION

This observational study will address the need for more high-quality data that examine the outcomes of RYGB and SG derived from the UK bariatric population. It will involve a comprehensive assessment and data collection at four study time points in the first year of surgery enabling an in-depth analysis of changes in body composition, PA levels, sedentary behaviour, physical function and strength, dietary intake, HRQoL and costs, relative to pre-surgery. Data collection will be carried out by using validated assessment methods and questionnaires. Another advantage of this study is the use of DXA scan, a reference standard to measure body composition[38]. Also, the use of accelerometer will generate high-quality data to measure objective PA levels and sedentary behaviour. Studies have shown that bariatric patients tend to over-report their PA levels when assessed using the coventional PA questionnaires[22].

Limitations of this study include a potential sample selection bias, due to the exclusion of patients with functional limitation (e.g. cognitive impairment, walking difficulties) and/or non-ambulatory

For peer review only - http://bmjopen.bmj.com/site/about/guidelines.xhtml

and patients with more than 200 kg of body weight owing to the weight limit of the DXA scan. Also, given resource limitations, only approximately 100 patients will be recruited in this one year observational cohort study. Nevertheless, this sample size is adequate to generate in-depth insights into the various outcomes of RYGB and SG.

CONCLUSION

BARI-LIFESTYLE observational study will produce a comprehensive data on the broad range of RYGB and SG outcomes derived from the UK bariatric population that is still scarce in the literature. The information gained from this study will inform future lifestyle programmes for post-bariatric patients.

Acknowledgements

We wish to thank Professor Rumana Omar for her input with regards to the statistical analysis plan. We gratefully acknowledge our Patient and Public Involvement group for their contribution to the study design as to ensure participants' acceptability. We would also like to thank all members of the Steering Committee and our research team at the Centre for Obesity Research, UCL for their invaluable inputs in the study.

Authors' contributions

RLB and FCJ designed the overall study and drafted the manuscript; AC coordinated the study to ensure GCP compliance; HK, JD and AK planned the assessment for dietary intake and HRQoL; BP and NL planned the assessment for PA levels, sedentary behaviour and physical function and strength; AGO advised on the statistical analysis plan; SM contributed to the analysis plan for healthcare resource utilisation and costs; AP and KC planned the assessment for body composition and review of comorbidities. RLB is the grant holder and chief investigator for the study. All authors

For peer review only

have contributed to the refinement of the study protocol and editing the manuscript. All authors have read and approved the final manuscript.

Funding

This study is supported by National Institute for Health Research (NIHR), the Sir Jules Thorn Charitable Trust and the Rosetrees Trust. The funders were not involved in decisions relating to the study design and data collection. They will not have any role in the study execution, analyses, interpretation of data nor in the writing of the manuscript and decision to submit results.

Competing interests

The authors declare that they have no conflict of interest.

REFERENCES

1. NICE. Obesity: Identification, assessment and management of overweight and obesity in children, young people and adults. London: NICE, 2014.
2. Ahmad A, Laverty AA, Aasheim E, et al. Eligibility for bariatric surgery among adults in England: analysis of a national cross-sectional survey. JRSM open 2014;5(1):2042533313512479.
3. Booth HP, Khan O, Fildes A, et al. Changing Epidemiology of Bariatric Surgery in the UK: Cohort Study Using Primary Care Electronic Health Records. Obes Surg 2016;26(8):1900-5.
4. Schneider J, Peterli R, Gass M, et al. Laparoscopic sleeve gastrectomy and Roux-en-Y gastric bypass lead to equal changes in body composition and energy metabolism 17 months postoperatively: a prospective randomized trial. Surg Obes Relat Dis 2016;12(3):563-70.
5. Nakamura KM, Haglind EG, Clowes JA, et al. Fracture risk following bariatric surgery: a population-based study. Osteoporosis international : a journal established as result of cooperation between the European Foundation for Osteoporosis and the National Osteoporosis Foundation of the USA 2014;25(1):151-8.
6. Lu CW, Chang YK, Chang HH, et al. Fracture Risk After Bariatric Surgery: A 12-Year Nationwide Cohort Study. Medicine 2015;94(48):e2087.
7. Liu C, Wu D, Zhang JF, et al. Changes in Bone Metabolism in Morbidly Obese Patients After Bariatric Surgery: A Meta-Analysis. Obes Surg 2016;26(1):91-7.
8. Yu EW. Bone metabolism after bariatric surgery. J Bone Miner Res 2014;29(7):1507-18.
9. Angrisani L, Santonicola A, Iovino P, et al. Bariatric Surgery Worldwide 2013. Obes Surg 2015;25(10):1822-32.
10. Pluskiewicz W, Buzga M, Holeczy P, et al. Bone mineral changes in spine and proximal femur in individual obese women after laparoscopic sleeve gastrectomy: a short-term study. Obes Surg 2012;22(7):1068-76.

For peer review only - http://bmjopen.bmj.com/site/about/guidelines.xhtml

11. Ruiz-Tovar J, Oller I, Priego P, et al. Short- and mid-term changes in bone mineral density after laparoscopic sleeve gastrectomy. Obes Surg 2013;23(7):861-6.

12. Carrasco F, Basfi-Fer K, Rojas P, et al. Changes in bone mineral density after sleeve gastrectomy or gastric bypass: relationships with variations in vitamin D, ghrelin, and adiponectin levels. Obes Surg 2014;24(6):877-84.

13. Mechanick JI, Youdim A, Jones DB, et al. Clinical practice guidelines for the perioperative nutritional, metabolic, and nonsurgical support of the bariatric surgery patient--2013 update: cosponsored by American Association of Clinical Endocrinologists, the Obesity Society, and American Society for Metabolic & Bariatric Surgery. Endocrine practice : official journal of the American College of Endocrinology and the American Association of Clinical Endocrinologists 2013;19(2):337-72.

14. Sheets CS, Peat CM, Berg KC, et al. Post-operative psychosocial predictors of outcome in bariatric surgery. Obes Surg 2015;25(2):330-45.

15. Sarwer DB, Wadden TA, Moore RH, et al. Preoperative eating behavior, postoperative dietary adherence, and weight loss after gastric bypass surgery. Surg Obes Relat Dis 2008;4(5):640-6.

16. Herman KM, Carver TE, Christou NV, et al. Keeping the weight off: physical activity, sitting time, and weight loss maintenance in bariatric surgery patients 2 to 16 years postsurgery. Obes Surg 2014;24(7):1064-72.

17. Bond DS, Phelan S, Wolfe LG, et al. Becoming physically active after bariatric surgery is associated with improved weight loss and health-related quality of life. Obesity (Silver Spring) 2009;17(1):78-83.

18. Campanha-Versiani L, Pereira DA, Ribeiro-Samora GA, et al. The Effect of a Muscle Weight-Bearing and Aerobic Exercise Program on the Body Composition, Muscular Strength, Biochemical Markers, and Bone Mass of Obese Patients Who Have Undergone Gastric Bypass Surgery. Obes Surg 2017.

19. Reid RE, Carver TE, Andersen KM, et al. Physical activity and sedentary behavior in bariatric patients long-term post-surgery. Obes Surg 2015;25(6):1073-7.

20. Biswas A, Oh PI, Faulkner GE, et al. Sedentary time and its association with risk for disease incidence, mortality, and hospitalization in adults: a systematic review and meta-analysis. Annals of internal medicine 2015;162(2):123-32.

21. O'Donovan G, Blazevich AJ, Boreham C, et al. The ABC of Physical Activity for Health: a consensus statement from the British Association of Sport and Exercise Sciences. J Sports Sci 2010;28(6):573-91.

22. Herring LY, Stevinson C, Davies MJ, et al. Changes in physical activity behaviour and physical function after bariatric surgery: a systematic review and meta-analysis. Obes Rev 2016;17(3):250-61.

23. Afshar S, Seymour K, Kelly SB, et al. Changes in physical activity after bariatric surgery: using objective and self-reported measures. Surg Obes Relat Dis 2017;13(3):474-83.

24. Raftopoulos I, Bernstein B, O'Hara K, et al. Protein intake compliance of morbidly obese patients undergoing bariatric surgery and its effect on weight loss and biochemical parameters. Surg Obes Relat Dis 2011;7(6):733-42.

25. Sherf Dagan S, Tovim TB, Keidar A, et al. Inadequate protein intake after laparoscopic sleeve gastrectomy surgery is associated with a greater fat free mass loss. Surg Obes Relat Dis 2017;13(1):101-09.

26. Moize V, Andreu A, Rodriguez L, et al. Protein intake and lean tissue mass retention following bariatric surgery. Clinical nutrition 2013;32(4):550-5.

27. Faria SL, Faria OP, Buffington C, et al. Dietary protein intake and bariatric surgery patients: a review. Obes Surg 2011;21(11):1798-805.

28. Ito MK, Goncalves VSS, Faria S, et al. Effect of Protein Intake on the Protein Status and Lean Mass of Post-Bariatric Surgery Patients: a Systematic Review. Obes Surg 2017;27(2):502-12.

For peer review only - http://bmjopen.bmj.com/site/about/guidelines.xhtml

29. Kolotkin RL, Meter K, Williams GR. Quality of life and obesity. Obes Rev 2001;2(4):219-29.
30. Munoz DJ, Lal M, Chen EY, et al. Why patients seek bariatric surgery: a qualitative and quantitative analysis of patient motivation. Obes Surg 2007;17(11):1487-91.
31. Testa MA, Simonson DC. Assessment of quality-of-life outcomes. N Engl J Med 1996;334(13):835-40.
32. Kolotkin RL, Andersen JR. A systematic review of reviews: exploring the relationship between obesity, weight loss and health-related quality of life. Clinical obesity 2017.
33. Lindekilde N, Gladstone BP, Lubeck M, et al. The impact of bariatric surgery on quality of life: a systematic review and meta-analysis. Obes Rev 2015;16(8):639-51.
34. Pollock RF, Chilcott J, Muduma G, et al. Laparoscopic adjustable gastric banding vs standard medical management in obese patients with type 2 diabetes: a budget impact analysis in the UK. Journal of medical economics 2013;16(2):249-59.
35. Terranova L, Busetto L, Vestri A, et al. Bariatric surgery: cost-effectiveness and budget impact. Obes Surg 2012;22(4):646-53.
36. Ackroyd R, Mouiel J, Chevallier JM, et al. Cost-effectiveness and budget impact of obesity surgery in patients with type-2 diabetes in three European countries. Obes Surg 2006;16(11):1488-503.
37. Bush K, Kivlahan DR, McDonell MB, et al. The AUDIT alcohol consumption questions (AUDIT-C): an effective brief screening test for problem drinking. Ambulatory Care Quality Improvement Project (ACQUIP). Alcohol Use Disorders Identification Test. Archives of internal medicine 1998;158(16):1789-95.
38. Lee SY, Gallagher D. Assessment methods in human body composition. Current opinion in clinical nutrition and metabolic care 2008;11(5):566-72.
39. Faria SL, Faria OP, Cardeal MD, et al. Validation study of multi-frequency bioelectrical impedance with dual-energy X-ray absorptiometry among obese patients. Obes Surg 2014;24(9):1476-80.
40. Migueles JH, Cadenas-Sanchez C, Ekelund U, et al. Accelerometer Data Collection and Processing Criteria to Assess Physical Activity and Other Outcomes: A Systematic Review and Practical Considerations. Sports medicine 2017.
41. A. T. S. Committee on Proficiency Standards for Clinical Pulmonary Function Laboratories. ATS statement: guidelines for the six-minute walk test. Am J Respir Crit Care Med 2002;166(1):111-7.
42. Pataky Z, Armand S, Muller-Pinget S, et al. Effects of obesity on functional capacity. Obesity (Silver Spring) 2014;22(1):56-62.
43. Sousa-Santos AR, Amaral TF. Differences in handgrip strength protocols to identify sarcopenia and frailty - a systematic review. BMC geriatrics 2017;17(1):238.
44. Yang YJ, Kim MK, Hwang SH, et al. Relative validities of 3-day food records and the food frequency questionnaire. Nutrition research and practice 2010;4(2):142-8.
45. Brooks R. EuroQol: the current state of play. Health policy 1996;37(1):53-72.
46. Dolan P. Modeling valuations for EuroQol health states. Medical care 1997;35(11):1095-108.
47. Kolotkin RL, Crosby RD, Kosloski KD, et al. Development of a brief measure to assess quality of life in obesity. Obes Res 2001;9(2):102-11.
48. Beck AT, Steer RA, Brown GK. Manual for the Beck Depression Inventory-II. San Antonio, TX, 1996.
49. Beecham J, Knapp M. Costing psychiatric interventions, in G. Thornicroft (ed.) Measuring Mental Health Needs. 2nf edition ed: Gaskell, 2001.
50. Curtis L, Burns A. Unit Costs of Health and Social Care 2015: Personal Social Services Research Unit, University of Kent, Canterbury, 2015.
51. Department of Health. National Schedule of Reference Costs: DOH, London, 2015.
52. British National Formulary. https://www.bnf.org/.

For peer review only

For peer review only - http://bmjopen.bmj.com/site/about/guidelines.xhtml

53. Barber J, Thompson S. Multiple regression of cost data: use of generalised linear models. J Health Serv Res Policy 2004;9(4):197-204.

Figure

Figure 1 Flow diagram of participant enrolment, consent, assessment and associated timeline.

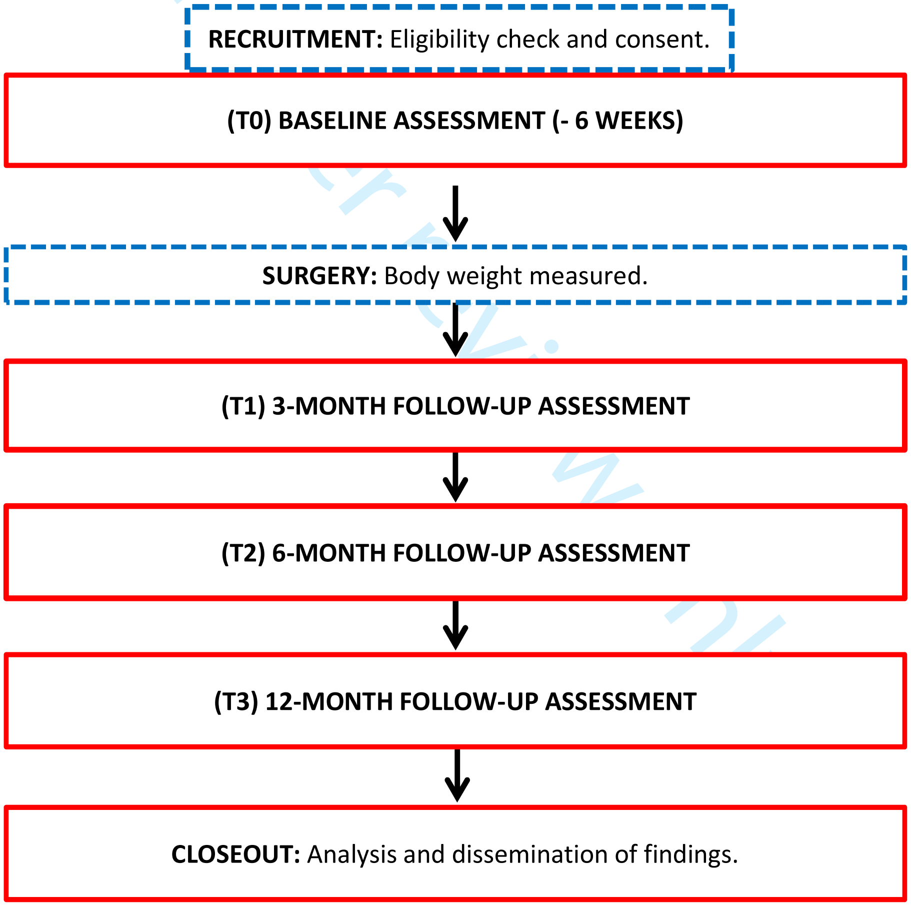

For peer review only - http://bmjopen.bmj.com/site/about/guidelines.xhtml

Table

Table 1 Participant eligibility criteria for participation in the BARI-LIFESTYLE observational study.

**INCLUSION CRITERIA**	
1	Adult aged between 18 to 65 years.
2	Planned to undergo either primary RYGB or primary SG surgery and fulfilling NICE eligibility criteria for bariatric surgery[1].
3	Able to read and write in English.
4	Willing and able to provide written informed consent.
5	Able to comply with study protocol.
6	Willing and able to wear a Fitbit wrist-based activity tracker device and an ActiGraph device.
**EXCLUSION CRITERIA**	
1	More than 200 kg of body weight due to the limitation of DXA scan.
2	Non-ambulatory.
3	Functional limitation.

For peer review only - http://bmjopen.bmj.com/site/about/guidelines.xhtml

For peer review only

For peer review only - http://bmjopen.bmj.com/site/about/guidelines.xhtml

Table 2 Study timeline and investigations.

	Baseline (T0)	Day of surgery	3-month post-surgery (T1)	6-month post-surgery (T2)	12-month post-surgery (T3)
Sociodemographic data	✓				
Height	✓				
Weight	✓	✓	✓	✓	✓
Blood pressure and heart rate	✓		✓	✓	✓
Dual-energy X-ray absorptiometry scan	✓				✓
Bioelectrical impedance analysis	✓		✓	✓	✓
Laboratory test	✓		✓	✓	✓
Physical activity levels (ActiGraph) and activity diary	✓		✓	✓	✓
Physical function and strength:					
6-minute walk test (6MWT)	✓		✓	✓	✓
Sit-to-stand (STS) test	✓		✓	✓	✓
Handgrip test	✓		✓	✓	✓
Dietary intake (3-day food diary)	✓		✓	✓	✓
Completion of questionnaires:					
EuroQol-5D-3L (EQ-5D-3L)	✓		✓	✓	✓
Impact of weight on Quality of Life-Lite (IWQOL-Lite)	✓		✓	✓	✓
Beck Depression Inventory-II (BDI-II)	✓		✓	✓	✓
Client Service Receipt Inventory (CSRI)	✓		✓	✓	✓
Review of medication	✓		✓	✓	✓
Review of comorbidities	✓		✓	✓	✓

For peer review only

For peer review only - http://bmjopen.bmj.com/site/about/guidelines.xhtml

SPIRIT
STANDARD PROTOCOL ITEMS: RECOMMENDATIONS FOR INTERVENTIONAL TRIALS

SPIRIT 2013 Checklist: Recommended items to address in a clinical trial protocol and related documents*

Section/item	Item No	Description
**Administrative information**		
Title	1	Descriptive title identifying the study design, population, interventions, and, if applicable, trial acronym **Page 1**
Trial registration	2a	Trial identifier and registry name. If not yet registered, name of intended registry **N/A**
	2b	All items from the World Health Organization Trial Registration Data Set **N/A**
Protocol version	3	Date and version identifier **N/A**
Funding	4	Sources and types of financial, material, and other support **Page 16**
Roles and responsibilities	5a	Names, affiliations, and roles of protocol contributors **Page 1 and page 15**
	5b	Name and contact information for the trial sponsor **N/A**
	5c	Role of study sponsor and funders, if any, in study design; collection, management, analysis, and interpretation of data; writing of the report; and the decision to submit the report for publication, including whether they will have ultimate authority over any of these activities **Page 16**
	5d	Composition, roles, and responsibilities of the coordinating centre, steering committee, endpoint adjudication committee, data management team, and other individuals or groups overseeing the trial, if applicable (see Item 21a for data monitoring committee) **N/A**

For peer review only

Introduction

Background and rationale	6a	Description of research question and justification for undertaking the trial, including summary of relevant studies (published and unpublished) examining benefits and harms for each intervention<br>**Page 4**
	6b	Explanation for choice of comparators<br>**N/A**
Objectives	7	Specific objectives or hypotheses<br>**Page 6**
Trial design	8	Description of trial design including type of trial (eg, parallel group, crossover, factorial, single group), allocation ratio, and framework (eg, superiority, equivalence, noninferiority, exploratory)<br>**Page 7**

Methods: Participants, interventions, and outcomes

Study setting	9	Description of study settings (eg, community clinic, academic hospital) and list of countries where data will be collected. Reference to where list of study sites can be obtained<br>**Page 7**
Eligibility criteria	10	Inclusion and exclusion criteria for participants. If applicable, eligibility criteria for study centres and individuals who will perform the interventions (eg, surgeons, psychotherapists)<br>**Page 20 (Table 1)**
Interventions	11a	Interventions for each group with sufficient detail to allow replication, including how and when they will be administered<br>**N/A**
	11b	Criteria for discontinuing or modifying allocated interventions for a given trial participant (eg, drug dose change in response to harms, participant request, or improving/worsening disease)<br>**N/A**
	11c	Strategies to improve adherence to intervention protocols, and any procedures for monitoring adherence (eg, drug tablet return, laboratory tests)<br>**N/A**
	11d	Relevant concomitant care and interventions that are permitted or prohibited during the trial<br>**N/A**

Outcomes	12	Primary, secondary, and other outcomes, including the specific measurement variable (eg, systolic blood pressure), analysis metric (eg, change from baseline, final value, time to event), method of aggregation (eg, median, proportion), and time point for each outcome. Explanation of the clinical relevance of chosen efficacy and harm outcomes is strongly recommended<br>**Page 9**
Participant timeline	13	Time schedule of enrolment, interventions (including any run-ins and washouts), assessments, and visits for participants. A schematic diagram is highly recommended (see Figure)<br>**Page 19 (Figure 1) and Page 21 (Table 2)**
Sample size	14	Estimated number of participants needed to achieve study objectives and how it was determined, including clinical and statistical assumptions supporting any sample size calculations<br>**Page 7 and page 15**
Recruitment	15	Strategies for achieving adequate participant enrolment to reach target sample size<br>**Page 8**

Methods: Assignment of interventions (for controlled trials)

Allocation:

Sequence generation	16a	Method of generating the allocation sequence (eg, computer-generated random numbers), and list of any factors for stratification. To reduce predictability of a random sequence, details of any planned restriction (eg, blocking) should be provided in a separate document that is unavailable to those who enrol participants or assign interventions<br>**N/A**
Allocation concealment mechanism	16b	Mechanism of implementing the allocation sequence (eg, central telephone; sequentially numbered, opaque, sealed envelopes), describing any steps to conceal the sequence until interventions are assigned<br>**N/A**
Implementation	16c	Who will generate the allocation sequence, who will enrol participants, and who will assign participants to interventions<br>**N/A**
Blinding (masking)	17a	Who will be blinded after assignment to interventions (eg, trial participants, care providers, outcome assessors, data analysts), and how<br>**N/A**

	17b	If blinded, circumstances under which unblinding is permissible, and procedure for revealing a participant's allocated intervention during the trial<br>**N/A**
**Methods: Data collection, management, and analysis**		
Data collection methods	18a	Plans for assessment and collection of outcome, baseline, and other trial data, including any related processes to promote data quality (eg, duplicate measurements, training of assessors) and a description of study instruments (eg, questionnaires, laboratory tests) along with their reliability and validity, if known. Reference to where data collection forms can be found, if not in the protocol<br>**Page 9 and 21 (Table 2)**
	18b	Plans to promote participant retention and complete follow-up, including list of any outcome data to be collected for participants who discontinue or deviate from intervention protocols<br>**N/A**
Data management	19	Plans for data entry, coding, security, and storage, including any related processes to promote data quality (eg, double data entry; range checks for data values). Reference to where details of data management procedures can be found, if not in the protocol<br>**Page 13**
Statistical methods	20a	Statistical methods for analysing primary and secondary outcomes. Reference to where other details of the statistical analysis plan can be found, if not in the protocol<br>**Page 12**
	20b	Methods for any additional analyses (eg, subgroup and adjusted analyses)<br>**Page 12**
	20c	Definition of analysis population relating to protocol non-adherence (eg, as randomised analysis), and any statistical methods to handle missing data (eg, multiple imputation)<br>**Page 13**
**Methods: Monitoring**		
Data monitoring	21a	Composition of data monitoring committee (DMC); summary of its role and reporting structure; statement of whether it is independent from the sponsor and competing interests; and reference to where further details about its charter can be found, if not in the protocol. Alternatively, an explanation of why a DMC is not needed<br>**N/A**

	21b	Description of any interim analyses and stopping guidelines, including who will have access to these interim results and make the final decision to terminate the trial **N/A**
Harms	22	Plans for collecting, assessing, reporting, and managing solicited and spontaneously reported adverse events and other unintended effects of trial interventions or trial conduct **Page 14**
Auditing	23	Frequency and procedures for auditing trial conduct, if any, and whether the process will be independent from investigators and the sponsor **N/A**
**Ethics and dissemination**		
Research ethics approval	24	Plans for seeking research ethics committee/institutional review board (REC/IRB) approval **Page 13**
Protocol amendments	25	Plans for communicating important protocol modifications (eg, changes to eligibility criteria, outcomes, analyses) to relevant parties (eg, investigators, REC/IRBs, trial participants, trial registries, journals, regulators) **N/A**
Consent or assent	26a	Who will obtain informed consent or assent from potential trial participants or authorised surrogates, and how (see Item 32) **Page 8**
	26b	Additional consent provisions for collection and use of participant data and biological specimens in ancillary studies, if applicable **N/A**
Confidentiality	27	How personal information about potential and enrolled participants will be collected, shared, and maintained in order to protect confidentiality before, during, and after the trial **Page 13**
Declaration of interests	28	Financial and other competing interests for principal investigators for the overall trial and each study site **Page 16**
Access to data	29	Statement of who will have access to the final trial dataset, and disclosure of contractual agreements that limit such access for investigators **Page 13**

For peer review only

Ancillary and post-trial care	30	Provisions, if any, for ancillary and post-trial care, and for compensation to those who suffer harm from trial participation<br>**N/A**
Dissemination policy	31a	Plans for investigators and sponsor to communicate trial results to participants, healthcare professionals, the public, and other relevant groups (eg, via publication, reporting in results databases, or other data sharing arrangements), including any publication restrictions<br>**Page 13**
	31b	Authorship eligibility guidelines and any intended use of professional writers<br>**N/A**
	31c	Plans, if any, for granting public access to the full protocol, participant-level dataset, and statistical code<br>**N/A**
**Appendices**		
Informed consent materials	32	Model consent form and other related documentation given to participants and authorised surrogates<br>**N/A**
Biological specimens	33	Plans for collection, laboratory evaluation, and storage of biological specimens for genetic or molecular analysis in the current trial and for future use in ancillary studies, if applicable<br>**N/A**

*It is strongly recommended that this checklist be read in conjunction with the SPIRIT 2013 Explanation & Elaboration for important clarification on the items. Amendments to the protocol should be tracked and dated. The SPIRIT checklist is copyrighted by the SPIRIT Group under the Creative Commons "Attribution-NonCommercial-NoDerivs 3.0 Unported" license.

For peer review only

Protocol for a one-year prospective, longitudinal cohort study of patients undergoing Roux-en-Y gastric bypass and sleeve gastrectomy: the BARI-LIFESTYLE observational study.

Journal:	*BMJ Open*
Manuscript ID	bmjopen-2017-020659.R1
Article Type:	Protocol
Date Submitted by the Author:	05-Jan-2018
Complete List of Authors:	Jassil, Friedrich; University College London, Centre for Obesity Research, Division of Medicine; University College London Hospitals, Bariatric Centre for Weight Management and Metabolic Surgery<br>Carnemolla, Alisia; University College London, Centre for Obesity Research, Division of Medicine; National Institute of Health Research, University College London Hospitals Biomedical Research Centre<br>Kingett, Helen; University College London Hospitals, Bariatric Centre for Weight Management and Metabolic Surgery; National Institute of Health Research, University College London Hospitals Biomedical Research Centre<br>Paton, Bruce; Institute of Sport, Exercise and Health<br>O'Keeffe, Aidan; University College London, Department of Statistical Science<br>Doyle, Jacqueline; University College London Hospitals, Bariatric Centre for Weight Management and Metabolic Surgery; National Institute of Health Research, University College London Hospitals Biomedical Research Centre<br>Morris, Stephen; University College London, Department of Applied Health Research<br>Lewis, Neville; University College London, The Hatter Cardiovascular Institute, Institute of Cardiovascular Science<br>Kirk, Amy; University College London Hospitals, Bariatric Centre for Weight Management and Metabolic Surgery; National Institute of Health Research, University College London Hospitals Biomedical Research Centre<br>Pucci, Andrea; University College London Hospitals, Bariatric Centre for Weight Management and Metabolic Surgery; University College London, Centre for Obesity Research, Division of Medicine<br>Chaiyasoot, Kusuma; University College London, Centre for Obesity Research, Division of Medicine; University College London Hospitals, Bariatric Centre for Weight Management and Metabolic Surgery<br>Batterham, Rachel; University College London, Centre for Obesity Research, Division of Medicine; University College London Hospitals, Bariatric Centre for Weight Management and Metabolic Surgery
**Primary Subject Heading**:	Surgery
Secondary Subject Heading:	Diabetes and endocrinology, Nutrition and metabolism, Gastroenterology and hepatology
Keywords:	Obesity, Bariatric Surgery, Body Composition, Physical Activity, Diet,

	Quality of Life

SCHOLARONE™
Manuscripts

For peer review only

Protocol for a one-year prospective, longitudinal cohort study of patients undergoing Roux-en-Y gastric bypass and sleeve gastrectomy: the BARI-LIFESTYLE observational study.

Friedrich C. Jassil [1,2], Alisia Carnemolla [1,3], Helen Kingett [2,3], Bruce Paton [4], Aidan G. O'Keeffe [5], Jaqueline Doyle [2,3] , Stephen Morris [6], Neville Lewis [7], Amy Kirk [2,3], Andrea Pucci [1,2], Kusuma Chaiyasoot [1,2] and Rachel L. Batterham [1,2,3*]

Author affiliations

[1] Centre for Obesity Research, Division of Medicine, University College London, London, United Kingdom

[2] University College London Hospitals, Bariatric Centre for Weight Management and Metabolic Surgery, London, United Kingdom

[3] National Institute of Health Research University College London Hospitals Biomedical Research Centre, London, United Kingdom

[4] Institute of Sport, Exercise and Health, London, United Kingdom

[5] Department of Statistical Science, University College London, London, United Kingdom

[6] Department of Applied Health Research, University College London, London, United Kingdom

[7] The Hatter Cardiovascular Institute, Institute of Cardiovascular Science, University College London, London, United Kingdom

*Corresponding author: Professor Rachel L. Batterham; r.batterham@ucl.ac.uk

Address: Centre for Obesity Research
Division of Medicine
University College London
Rayne Building, 5 University Street
London WC1E 6JF, United Kingdom

For peer review only - http://bmjopen.bmj.com/site/about/guidelines.xhtml

ABSTRACT

Introduction: Roux-en-Y gastric bypass and sleeve gastrectomy are the two most common bariatric surgery performed in the UK that result in comparable weight loss and remission of obesity-associated co-morbidities. However, there is a paucity of studies examining the impact of these procedures upon body composition, physical activity levels, sedentary behaviour, physical function and strength, dietary intake, health-related quality of life and costs.

Methods and analysis: The BARI-LIFESTYLE observational study is a one-year prospective, longitudinal cohort study within a real-world routine clinical care setting aiming to recruit 100 patients with severe obesity undergoing either primary Roux-en-Y gastric bypass or sleeve gastrectomy from two bariatric centres in London, UK. Participants will be followed-up four times during the study period; pre-surgery baseline (T0) and at 3 (T1), 6 (T2), and 12-month (T3) post-surgery. In addition to the standard follow-up investigations, assessments including dual-energy X-ray absorptiometry scan, bioelectric impedance analysis, 6-minute walk test, sit-to-stand test, and handgrip test will be undertaken together with completion of questionnaires. Physical activity levels and sedentary behaviour will be assessed using accelerometer, and dietary intake will be recorded using a 3-day food diary. Outcome measures will include: body weight, body fat mass, lean muscle mass, bone mineral density, physical activity levels, sedentary behaviour, physical function and strength, dietary intake, health-related quality of life, remission of co-morbidities, healthcare resource utilisation and costs.

Ethics and dissemination: This study has been reviewed and given a favourable ethical opinion by London-Dulwich Research Ethics Committee (17/LO/0950). The results will be presented to stakeholder groups locally, nationally and internationally and published in peer-reviewed medical journals. The lay-person summary of the findings will be published on the Centre for Obesity Research, University College London website (http://www.ucl.ac.uk/obesity).

For peer review only - http://bmjopen.bmj.com/site/about/guidelines.xhtml

Strengths and limitations of this study

- A comprehensive prospective, longitudinal study with detailed assessments undertaken prior to and for one year following bariatric surgery examining changes in body composition, physical activity (PA) levels, sedentary behaviour, physical function and strength, dietary intake, health-related quality of life and costs, relative to baseline pre-surgery.
- The use of validated research tools (accelerometer to assess PA levels and sedentary behaviour, dual-energy X-ray absorptiometry [DXA] scan to assess body composition and validated questionnaires) will generate high-quality data.
- The study design does not include a conventional intensive lifestyle intervention (non-surgical) as a comparator group and patients will not be randomised to RYGB or SG in order to reflect current real-world clinical care.
- A potential sample selection bias due to exclusion of patients with functional limitation and/or non-ambulatory and patients with more than 200 kg of body weight owing to the weight limit of the DXA scan.
- A relatively small sample size, nevertheless, this number is adequate to generate in-depth insights into the various outcomes of Roux-en-Y gastric bypass and sleeve gastrectomy as delivered in the UK healthcare setting.

For peer review only

INTRODUCTION

Bariatric surgery engenders marked sustained weight loss and is recommended by the National Institute for Health and Care Excellence (NICE) as a treatment option for people of severe obesity[1], estimated to affect approximately 2.6 million adults in the UK[2]. Roux-en-Y gastric bypass (RYGB) and sleeve gastrectomy (SG) are now the two most common procedures performed in the UK, which result in comparable weight loss and remission of obesity associated co-morbidities[3]. However, there is a paucity of studies examining the impact of these procedures upon body composition, particularly bone mineral density (BMD), physical activity (PA) levels, sedentary behaviour, physical function and strength, dietary intake, health-related quality of life (HRQoL) and costs. Furthermore, current eligibility and success criteria of bariatric surgery are mainly based on body weight, body mass index (BMI) and excess weight loss but evidence have shown various beneficial outcomes of the surgery above and beyond weight loss alone, hence highlighting the need for more functional pre- and post-operative patient assessment[4 5].

Bariatric surgery leads to a marked decrease in fat mass (FM), but fat free mass (FFM) particularly bone mass is also reduced post-surgery[6], potentially negatively impacting on physical function and strength, and putting patients at increased risk of osteoporotic fracture in the future[7 8]. Moreover, a recent study has revealed a positive association between changes in adiposity with cardiometabolic outcomes post-surgery, indicating the usefulness of incorporating body composition assessment[9]. Surgical modification of the gastrointestinal tract impairs the intake and/or absorption of essential nutrients for bone health that consequently perturbs bone metabolism, leading to BMD deterioration[7 8 10 11]. Significant bone mass loss has been reported to occur rapidly in the first year of surgery and continues to deteriorate up to 3 years even after maximum weight loss has been achieved[10]. However, these data, are mainly based on studies undertaken in patients who underwent RYGB whereas SG is now the most common procedure undertaken both in the UK and

For peer review only

For peer review only - http://bmjopen.bmj.com/site/about/guidelines.xhtml

globally[3 12]. Currently, it is unclear whether the rate of bone mass loss following SG paralells weight loss[13-15]. Given that the number of younger patients and women of childbearing age undergoing bariatric surgery continues to increase and BMD measurement is not a routine follow-up investigation[16], there is an urgent need to assess the impact of RYBG and SG on bone health in the UK bariatric population.

Adherence to a post-bariatric lifestyle changes is the cornerstone of a successful weight loss[17]. Studies have shown that greater PA, lower sedentary time and high compliance to dietary recommendation post-surgery associate with greater weight loss and FM loss, preservation of lean muscle mass (LMM) and bone mass, as well as improvement in HRQoL[18-22]. However, patients spend 80% of their waking time in sedentary behaviour post-surgery[23], activity that associates with increased risk of cardiometabolic disease and mortality[24]. [22]Following surgery, patients are advised to undertake at least 150 minutes of moderate-to-vigorous physical activity (MVPA) per week, a duration and intensity that are recommended to reap the metabolic benefit of PA[25]. However, objectively measured MVPA decreases post-surgery with only 10% of patients achieving the recommended MVPA levels[26]. Likewise, a recent study undertaken in the UK has reported that weight loss post-surgery did not correspond to improvement in MVPA and sedentary behaviour. However, the small sample size of this study (n=22) together with relatively short follow-up period limited its generalisability[27]. Further studies are therefore required to expand the information in this regard. In terms of dietary recommendations, daily protein intake of 60 g or more post-surgery is crucial for increasing satiety, preserving LMM, improving body composition and preventing against weight regain[28-31]. However, most patients are unable to achieve this in the first postoperative year, the period when rapid weight loss occurs[32]. Whether this is also the case for UK bariatric population is not known as no such data has ever been reported thus far[32].

For peer review only - http://bmjopen.bmj.com/site/about/guidelines.xhtml

Impaired HRQoL is common in obesity[33] and often one of the driving factors for seeking weight loss surgery[34]. HRQoL is defined as individuals' perception of well-being that refers to physical, psychological and social domains of health[35]. Most studies reported improvement in all HRQoL domains with greater scores observed in the first post-operative year although some studies showed that the improvement is limited to only the physical domain but not the mental health component of HRQoL[36]. Despite mounting evidence in the international literature reporting the beneficial impact of bariatric surgery on HRQoL, data from the UK bariatric population does not exist[37]. There is some evidence that bariatric surgery can reduce in cost savings that offset the initial costs of surgery, though little UK evidence for RYGB and SG[38-40].

Taken together the lack of post-operative data coupled with recommendations from systematic reviews[26 32] provide a strong rational to undertake a prospective study to evaluate the impact of RYGB and SG upon body composition particularly BMD, PA levels, sedentary behaviour, physical function and strength, dietary intake, HRQoL and costs in a UK bariatric population. Information gained from this study will provide valuable data to inform the implementation of future post-surgery lifestyle programmes with the aim of maximising the beneficial outcomes of bariatric surgery as highlighted by NICE[1]. This paper details the study design and outcomes of the BARI-LIFESTYLE observational study.

OBJECTIVES

The overall objective of BARI-LIFESTYLE observational study is to evaluate the impact of RYGB and SG on changes in body weight, body composition, PA levels, sedentary behaviour, physical function and strength, dietary intake, HRQoL, remission of co-morbidities, healthcare resource utilisation and costs in a cohort of 100 patients.

The specific objectives are to evaluate post-surgery changes in:

For peer review only - http://bmjopen.bmj.com/site/about/guidelines.xhtml

1. Percentage weight loss (%WL) at one year post-surgery, relative to baseline pre-surgery weight.
2. Body fat mass, assessed using dual-energy X-ray absorptiometry (DXA) scan and bioelectrical impedance analysis (BIA), relative to pre-surgery at 12 months post-surgery.
3. LMM, assessed using DXA scan and BIA, relative to pre-surgery at 12 months post-surgery.
4. BMD, assessed using DXA scan and BIA, relative to pre-surgery at 12 months post-surgery.
5. PA levels (light, moderate, vigorous), percentage achieving 150 minutes of MVPA in a week and sedentary behaviour assessed using accelerometer at 3, 6, and 12 months post-surgery, relative to pre-surgery.
6. Physical function and strength assessed using 6-minute walk test (6MWT), sit-to-stand (STS) test and handgrip test at 3, 6 and 12 months post-surgery, relative to pre-surgery.
7. Dietary intake assessed using food diary at 3, 6 and 12 months post-surgery, relative to pre-surgery.
8. HRQoL assessed using EuroQol-5D-3L (EQ-5D-3L) and Impact of Weight on Quality of Life-Lite (IWQOL-Lite) at 3, 6 and 12 months post-surgery, relative to pre-surgery.
9. Characteristics of attitude and symptoms of depression assessed using Beck Depression Inventory-II (BDI-II) at 3, 6 and 12 months post-surgery, relative to pre-surgery.
10. Obesity-associated comorbidities (type 2 diabetes [T2D], dyslipidaemia, hypertension, obstructive sleep apnoea [OSA]) at 3, 6 and 12 months post-surgery, relative to pre-surgery.
11. Healthcare resource utilisation and costs assessed using an adapted version of the Client Service Receipt Inventory (CSRI) at 3, 6 and 12 months post-surgery, relative to pre-surgery.

METHODS AND ANALYSIS

Study design and setting

BARI-LIFESTYLE observational study is a prospective, longitudinal cohort study within routine clinical care setting of patients undergoing bariatric surgery conducted in London, UK (Figure 1). A total of

For peer review only

For peer review only - http://bmjopen.bmj.com/site/about/guidelines.xhtml

100 patients who are planned to undergo either primary RYGB or SG will be recruited over a 2-year period from 2018 to 2019, and will be followed for up to 12 months post-surgery. Recruitment will take place at the Bariatric Centre for Weight Management and Metabolic Surgery, University College London Hospitals (UCLH) (study site) and the Bariatric and Obesity Surgery Clinic at the Whittington Hospital that acts as a participant identification centre (PIC). Participants recruited from the Whittington Hospital will have their surgical procedure undertaken at the same centre, but all study procedures such as written informed consent, baseline assessment, post-surgery follow-up care and study assessments will be undertaken by the bariatric team at UCLH. In both centres, the decision for procedure selection is based on informed patient preference after standardised counselling including details, potential risks, and benefits of each procedure that adheres to the current international guideline for the surgical recommendation for obesity and weight-related disease[41]. This study is carried out by the Centre for Obesity Research, Division of Medicine, University College London (UCL), with an expected total duration of 36 months, from the first participant enrolled to last participant follow-up.

Participants and recruitment

Patients will be screened for suitability for the study by the bariatric team at the study site and PIC based on the inclusion and exclusion criteria when they attend the standard pre-surgical assessment (Table 1). Verbal consent will be sought from those fulfilling the eligibility criteria and interested in participating to be approached by a research investigator. Patients will be given a participant information sheet inviting them to participate in a one-year prospective, longitudinal cohort study looking at the effect of bariatric surgery on body weight, body composition, PA levels, sedentary behaviour, physical function and strength, dietary intake, HRQoL, remission of co-morbidities, healthcare resource utilisation and costs. Consented participants will then be scheduled to attend a baseline assessment, approximately 6 weeks before surgery day at the study site. Each participant will be given a Fitbit Alta HR to enable them to self-monitor their activity levels and to reduce

For peer review only - http://bmjopen.bmj.com/site/about/guidelines.xhtml

sedentary behaviour. Based on the weekly number of bariatric procedures undertaken at UCLH and Whittington Hospital and after considering the eligibility criteria, estimated recruitment rate is approximately 7 participants per month. Hence, the expected recruitment period for the study is 15 to 20 months.

All participants will receive the standardised post-bariatric care as stipulated by NICE[1]. Participants will attend the study site for monitoring of nutritional intake, vitamin and mineral deficiencies, comorbidities and medication review. Participants will receive verbal PA and dietary advice from a specialist nurse and dietitian at weeks 12 and 36 post-surgery, respectively.

Outcomes measures

Outcome measures will be collected at four study time points, designed to coincide with normal follow-up care visits; baseline visit at approximately 6 weeks before surgery (T0) then at 3 (T1), 6 (T2), and 12-month (T3) post-surgery (Table 2).

Sociodemographic, medical history, and physical examination

Participants' sociodemographic data, medical history, and physical examination will be completed by the bariatric team at the baseline visit. Data to be captured including age, gender, ethnicity, educational level, marital status, medication intake, weight history, pregnancy history, alcohol consumption using the AUDIT-C questionnaire[42], smoking habits and family history of obesity and comorbidities.

Primary outcome

Body weight will be measured using a weighing scale (Model VT200/220, Vishay Transducers Ltd., CA, USA) with participants wearing light clothes and without shoes and heavy accessories, to the nearest 0.1 kg. Similarly, height will be determined using a stadiometer (Seca 242, Seca GmbH & Co.

For peer review only - http://bmjopen.bmj.com/site/about/guidelines.xhtml

KG, Hamburg, Germany) to the nearest 0.01 m. %WL will be calculated using the following formula: %WL = [(weight on the day of surgery – weight at time-point after surgery)/weight on the day of surgery] × 100, measured at each study time point.

Secondary outcomes

Body composition (body fat mass, LMM and BMD)

Body composition will be assessed at baseline and 12-months post-surgery using DXA scan (Discovery™ A DXA system, software v13.4.2, Hologic®, Inc., MA, USA). DXA scan uses ionising radiation to measure different body compartments. This is the current reference standard for assessing body composition and a gold standard method to diagnose osteopenia and osteoporosis[43]. In addition, body composition will be measured using BIA (Tanita DC-430MAS, Tanita Corp., Tokyo, Japan) at each study visit. This is a non-invasive, easy to perform and cheaper option to measure body composition that is based on the differences in electrical conductivity of FM and FFM tissues[44].

PA levels and sedentary behaviour

PA and time spent in light, moderate and vigorous activities, and sedentary behaviour will be measured objectively using ActiGraph wGT3X-BT (Pensacola, FL, USA), an accelerometer-based activity monitor[45]. Participants will be instructed to wear the ActiGraph on their dominant hip for one week, from waking in the morning until going to bed at night, and to remove it only during water-based activities. Additionally, participants will be asked to keep an activity diary throughout the week, to assist interpretation of data from the device. Both the device and activity diary have to be returned to the investigators for data analysis (ActiLife software v6.13.3, Pensacola, FL, USA).

Physical function and strength

Participants' functional capacity will be assessed using a 6MWT, a self-paced, submaximal assessment of functional capacity used to prescribe appropriate exercise[46]. Lower body functional

For peer review only - http://bmjopen.bmj.com/site/about/guidelines.xhtml

capacity and strength will be assessed using the STS test[47]. Static muscle strength will be assessed using Jamar Hydraulic Hand Dynamometer (Petterson Medical, IL, USA)[48].

Dietary intake

All participants will be required to keep a 3-day food diary (2 working days and 1 weekend day) for one week at each study time point. This method has a higher agreement with the 9 days food dairy compared to the food frequency questionnaire[49] whilst reducing the burden to patients and thus promoting better compliance for documenting food intake. The completed food diary will be returned to the investigators together with the ActiGraph and activity diary by using a stamped addressed envelope provided to participants.

HRQoL

HRQoL will be assessed using EQ-5D-3L and IWQOL-Lite. The EQ-5D-3L descriptive system is a 5-item self-report questionnaire that assesses the following domains: mobility, self-care, usual activities, pain/discomfort and anxiety/depression, and a visual analogue scale, which records self-rated health on a 0 to 100 scale[50]. EQ-5D-3L health states will be converted into utility values using a formula that attaches weights to each level in each dimension based on valuations by general population samples. We will use a value set for the UK population to calculate utility values at each time point for every participant[51]. The IWQOL-Lite is a 31-item, self-report, obesity and overweight-specific measure of HRQoL[52]. This tool consists of a total score and scores on each of five scales – physical function, self-esteem, sexual life, public distress, and work; higher scores indicate better HRQoL.

Attitude and symptoms of depression

BDI-II is a 21-item self-report questionnaire that assesses mood over the past week[53]. Symptoms of depression are classified by the total score: minimal, mild, moderate, and severe symptoms.

For peer review only - http://bmjopen.bmj.com/site/about/guidelines.xhtml

Obesity-associated comorbidities

Co-morbidities (T2D, dyslipidaemia, hypertension, OSA) and medication review will be carried out at each study time point.

Healthcare resource utilisation and costs

Resource use data will be collected using an adapted version of the CSRI[54], including the costs of bariatric surgery plus pre-surgery visits, number of contacts with healthcare professionals, visits to specialist clinics, the emergency department, admissions to the hospital, primary care contacts, and medications. Resource use data will be converted into costs using published unit costs[55-57]. In addition, information regarding support from informal carers, employment status and time off work will be collected. Resource use data will be collected for the previous 6 months at the baseline visit and since participants' last study visit at each post-surgery study time point.

Sample size

A sample size of 100 patients will be enough to model the primary outcome and the range of secondary outcomes with a reasonable level precision and with regard to the number of patients who are likely to be recruited within the study's time frame. Also, a sample size of 100 patients will be sufficient to estimate the %WL at one year post-surgery to within ±2.5% using a 95% confidence interval. This calculation accounts for a possible drop-out rate of up to 25% and assumes a conservative estimate for standard deviation of %WL of 10%. This sample size should also ensure that there are enough data points for linear mixed effects models to be fitted with parameter estimates that have a satisfactory level of precision and where the model fitting algorithm will converge.

Statistical analysis

For peer review only

For peer review only - http://bmjopen.bmj.com/site/about/guidelines.xhtml

311 The demographic and medical history information collected at baseline shall be presented in a table. Categorical variables shall be reported as raw numbers and percentages. Reports of continuous variables shall include mean, median, range and standard deviation.

315 ***Primary outcome analysis***

316 The primary outcome is the %WL measured longitudinally at baseline and 12 months post-surgery. %WL will be analysed using a linear mixed effects model over the three post-surgery time points (3 months, 6 months and 12 months) after controlling for the baseline body weight measure and height. Model assumptions shall be checked and suitable transforms of the primary outcome variable considered if necessary. In addition, overall percentage change in weight since baseline shall be computed marginally at each of 3, 6 and 12 months and displayed graphically.

323 ***Secondary outcomes analyses***

324 Analyses of longitudinal secondary outcomes shall be performed using linear mixed effects regression models, with a normal distribution assumed for continuous outcomes (or a suitable transform of these outcomes). Model parameter estimates together with appropriate 95% confidence intervals shall be reported. Categorical outcomes (e.g. proportions of participants with co-morbidities) shall be summarised in tabular form at each time point. Where appropriate (for example, for proportions), estimates and 95% confidence intervals will be presented. To analyse costs, we will assume the costs measured at baseline for the preceding 6 months would persist during follow-up in the absence of surgery; we will then compare post-surgery costs with predicted costs that would have been incurred in the absence of surgery. To account for skewness of the cost data, we will use a generalised linear model with gamma family and log link[58].

335 ***Missing data***

For peer review only - http://bmjopen.bmj.com/site/about/guidelines.xhtml

Bias due to missing data will be investigated by comparing the baseline characteristics of participants with and without missing values. Depending on the extent of missingness, the predictors of missing values will be identified. The primary outcome analysis will be adjusted for those predictors of missing values, which are related to missingness. Multiple imputation using chained equations shall be considered as part of a sensitivity analysis for missing data in the primary outcome model.

Data storage and retention

All data will be handled in accordance with the UK Data Protection Act 1998. Physical data will be stored in a secure room with limited access to only members of the research team, whereas computers storing electronic data will be encrypted and password protected. Each participant will be given a unique study identification number and used on their records instead of their name. The master list linking participants' name and the study identification number will be kept in a secure location. This way, participants' personal identity and data collected in the study cannot be linked by anyone outside the study team. This study is registered with the UCL Data Protection (Reference: Z6364106/2017/04/43). At the end of the study, all essential documentation will be archived securely for a minimum of 20 years from the declaration of the end of study.

ETHICS AND DISSEMINATION

This study has been reviewed and given a favourable ethical opinion by London-Dulwich Research Ethics Committee (Reference: 17/LO/0950). Potential participants will be explained in detail regarding the aims, methods, anticipated advantages and disadvantages of participation in the study by Good Clinical Practice (GCP) trained investigators prior to obtaining their written informed consent. Participants will be informed that their participation is on a voluntary basis, and they have the right to withdraw from the study at any time without affecting their present and future medical care. No research procedures will be undertaken prior to patients giving written informed consent.

For peer review only - http://bmjopen.bmj.com/site/about/guidelines.xhtml

As a duty of care, all possible adverse events will be collected from the day participants consented for the study to monitor their safety.

The findings will be presented to stakeholder groups locally, nationally and internationally and published in peer-reviewed medical journals. The lay-person summary of the findings will be published on the Centre for Obesity Research, University College London website (http://www.ucl.ac.uk/obesity). The results will be fully anonymised, and none of the participants will be identified in any report or publication.

ADVANTAGE AND LIMITATION

This observational study will address the need for more high-quality data that examine the outcomes of RYGB and SG derived from the UK bariatric population. It will involve a comprehensive assessment and data collection at four study time points in the first year of surgery enabling an in-depth analysis of changes in body composition, PA levels, sedentary behaviour, physical function and strength, dietary intake, HRQoL and costs, relative to pre-surgery. Data collection will be carried out by using validated assessment methods and questionnaires. Another advantage of this study is the use of DXA scan, a reference standard to measure body composition[43]. Also, the use of accelerometer will generate high-quality data to measure objective PA levels and sedentary behaviour. Studies have shown that bariatric patients tend to over-report their PA levels when assessed using the coventional PA questionnaires[26].

This protocol for an observational study is not without limitations. First, the study design does not include a conventional intensive lifestyle intervention (non-surgical) as a comparator group. Second, patients will not be randomised for surgical procedure as this study does not aim to compare between RYGB and SG but aims to examine ‘real-world’ clinical outcomes where the patient/healthcare professional make an informed choice about which procedures is best. However,

For peer review only - http://bmjopen.bmj.com/site/about/guidelines.xhtml

data that will be generated from this study will allow us to power a subsequent randomised study. Third, a potential sample selection bias due to the exclusion of patients with functional limitation (e.g. cognitive impairment, walking difficulties) and/or non-ambulatory and patients with more than 200 kg of body weight owing to the weight limit of the DXA scan. Finally, given resource limitations, only approximately 100 patients will be recruited in this one year observational cohort study. Nevertheless, this sample size is adequate to generate in-depth insights into the various outcomes of RYGB and SG.

CONCLUSION

BARI-LIFESTYLE observational study will produce a comprehensive data on the broad range of RYGB and SG outcomes derived from the UK bariatric population that is still scarce in the literature. The information gained from this study will inform future lifestyle programmes for post-bariatric patients.

Acknowledgements

We wish to thank Professor Rumana Omar for her input with regards to the statistical analysis plan. We gratefully acknowledge our Patient and Public Involvement group for their contribution to the study design as to ensure participants' acceptability. We would also like to thank all members of the Steering Committee and our research team at the Centre for Obesity Research, UCL for their invaluable inputs in the study.

Authors' contributions

RLB and FCJ designed the overall study and drafted the manuscript; AC coordinated the study to ensure GCP compliance; HK, JD and AK planned the assessment for dietary intake and HRQoL; BP and NL planned the assessment for PA levels, sedentary behaviour and physical function and strength; AGO advised on the statistical analysis plan; SM contributed to the analysis plan for

For peer review only

For peer review only - http://bmjopen.bmj.com/site/about/guidelines.xhtml

healthcare resource utilisation and costs; AP and KC planned the assessment for body composition and review of comorbidities. RLB is the grant holder and chief investigator for the study. All authors have contributed to the refinement of the study protocol and editing the manuscript. All authors have read and approved the final manuscript.

Funding

This study is supported by National Institute for Health Research (NIHR), the Sir Jules Thorn Charitable Trust and the Rosetrees Trust. The funders were not involved in decisions relating to the study design and data collection. They will not have any role in the study execution, analyses, interpretation of data nor in the writing of the manuscript and decision to submit results.

Competing interests

The authors declare that they have no conflict of interest.

REFERENCES

1. NICE. Obesity: Identification, assessment and management of overweight and obesity in children, young people and adults. London: NICE 2014.
2. Ahmad A, Laverty AA, Aasheim E, et al. Eligibility for bariatric surgery among adults in England: analysis of a national cross-sectional survey. *JRSM open* 2014;5(1):2042533313512479. doi: 10.1177/2042533313512479
3. Booth HP, Khan O, Fildes A, et al. Changing Epidemiology of Bariatric Surgery in the UK: Cohort Study Using Primary Care Electronic Health Records. *Obes Surg* 2016;26(8):1900-5. doi: 10.1007/s11695-015-2032-9
4. Fruhbeck G. Bariatric and metabolic surgery: a shift in eligibility and success criteria. *Nature reviews Endocrinology* 2015;11(8):465-77. doi: 10.1038/nrendo.2015.84
5. Cummings DE, Cohen RV. Beyond BMI: the need for new guidelines governing the use of bariatric and metabolic surgery. *Lancet Diabetes Endocrinol* 2014;2(2):175-81. doi: 10.1016/S2213-8587(13)70198-0 [published Online First: 2014/03/14]
6. Schneider J, Peterli R, Gass M, et al. Laparoscopic sleeve gastrectomy and Roux-en-Y gastric bypass lead to equal changes in body composition and energy metabolism 17 months postoperatively: a prospective randomized trial. *Surg Obes Relat Dis* 2016;12(3):563-70. doi: 10.1016/j.soard.2015.07.002
7. Nakamura KM, Haglind EG, Clowes JA, et al. Fracture risk following bariatric surgery: a population-based study. *Osteoporosis international : a journal established as result of cooperation between the European Foundation for Osteoporosis and the National Osteoporosis Foundation of the USA* 2014;25(1):151-8. doi: 10.1007/s00198-013-2463-x

For peer review only

For peer review only - http://bmjopen.bmj.com/site/about/guidelines.xhtml

8. Lu CW, Chang YK, Chang HH, et al. Fracture Risk After Bariatric Surgery: A 12-Year Nationwide Cohort Study. *Medicine* 2015;94(48):e2087. doi: 10.1097/MD.0000000000002087

9. Gomez-Ambrosi J, Andrada P, Valenti V, et al. Dissociation of body mass index, excess weight loss and body fat percentage trajectories after 3 years of gastric bypass: relationship with metabolic outcomes. *Int J Obes (Lond)* 2017;41(9):1379-87. doi: 10.1038/ijo.2017.134 [published Online First: 2017/06/07]

10. Liu C, Wu D, Zhang JF, et al. Changes in Bone Metabolism in Morbidly Obese Patients After Bariatric Surgery: A Meta-Analysis. *Obes Surg* 2016;26(1):91-7. doi: 10.1007/s11695-015-1724-5

11. Yu EW. Bone metabolism after bariatric surgery. *J Bone Miner Res* 2014;29(7):1507-18. doi: 10.1002/jbmr.2226

12. Angrisani L, Santonicola A, Iovino P, et al. Bariatric Surgery Worldwide 2013. *Obes Surg* 2015;25(10):1822-32. doi: 10.1007/s11695-015-1657-z

13. Pluskiewicz W, Buzga M, Holeczy P, et al. Bone mineral changes in spine and proximal femur in individual obese women after laparoscopic sleeve gastrectomy: a short-term study. *Obes Surg* 2012;22(7):1068-76. doi: 10.1007/s11695-012-0654-8

14. Ruiz-Tovar J, Oller I, Priego P, et al. Short- and mid-term changes in bone mineral density after laparoscopic sleeve gastrectomy. *Obes Surg* 2013;23(7):861-6. doi: 10.1007/s11695-013-0866-6

15. Carrasco F, Basfi-Fer K, Rojas P, et al. Changes in bone mineral density after sleeve gastrectomy or gastric bypass: relationships with variations in vitamin D, ghrelin, and adiponectin levels. *Obes Surg* 2014;24(6):877-84. doi: 10.1007/s11695-014-1179-0

16. Mechanick JI, Youdim A, Jones DB, et al. Clinical practice guidelines for the perioperative nutritional, metabolic, and nonsurgical support of the bariatric surgery patient--2013 update: cosponsored by American Association of Clinical Endocrinologists, the Obesity Society, and American Society for Metabolic & Bariatric Surgery. *Endocrine practice : official journal of the American College of Endocrinology and the American Association of Clinical Endocrinologists* 2013;19(2):337-72. doi: 10.4158/EP12437.GL

17. Sheets CS, Peat CM, Berg KC, et al. Post-operative psychosocial predictors of outcome in bariatric surgery. *Obes Surg* 2015;25(2):330-45. doi: 10.1007/s11695-014-1490-9

18. Sarwer DB, Wadden TA, Moore RH, et al. Preoperative eating behavior, postoperative dietary adherence, and weight loss after gastric bypass surgery. *Surg Obes Relat Dis* 2008;4(5):640-6. doi: 10.1016/j.soard.2008.04.013

19. Herman KM, Carver TE, Christou NV, et al. Keeping the weight off: physical activity, sitting time, and weight loss maintenance in bariatric surgery patients 2 to 16 years postsurgery. *Obes Surg* 2014;24(7):1064-72. doi: 10.1007/s11695-014-1212-3

20. Bond DS, Phelan S, Wolfe LG, et al. Becoming physically active after bariatric surgery is associated with improved weight loss and health-related quality of life. *Obesity (Silver Spring)* 2009;17(1):78-83. doi: 10.1038/oby.2008.501

21. Campanha-Versiani L, Pereira DA, Ribeiro-Samora GA, et al. The Effect of a Muscle Weight-Bearing and Aerobic Exercise Program on the Body Composition, Muscular Strength, Biochemical Markers, and Bone Mass of Obese Patients Who Have Undergone Gastric Bypass Surgery. *Obes Surg* 2017 doi: 10.1007/s11695-017-2618-5

22. Vatier C, Henegar C, Ciangura C, et al. Dynamic relations between sedentary behavior, physical activity, and body composition after bariatric surgery. *Obes Surg* 2012;22(8):1251-6. doi: 10.1007/s11695-012-0619-y

23. Reid RE, Carver TE, Andersen KM, et al. Physical activity and sedentary behavior in bariatric patients long-term post-surgery. *Obes Surg* 2015;25(6):1073-7. doi: 10.1007/s11695-015-1624-8

For peer review only - http://bmjopen.bmj.com/site/about/guidelines.xhtml

24. Biswas A, Oh PI, Faulkner GE, et al. Sedentary time and its association with risk for disease incidence, mortality, and hospitalization in adults: a systematic review and meta-analysis. *Annals of internal medicine* 2015;162(2):123-32. doi: 10.7326/M14-1651

25. O'Donovan G, Blazevich AJ, Boreham C, et al. The ABC of Physical Activity for Health: a consensus statement from the British Association of Sport and Exercise Sciences. *J Sports Sci* 2010;28(6):573-91. doi: 10.1080/02640411003671212

26. Herring LY, Stevinson C, Davies MJ, et al. Changes in physical activity behaviour and physical function after bariatric surgery: a systematic review and meta-analysis. *Obes Rev* 2016;17(3):250-61. doi: 10.1111/obr.12361

27. Afshar S, Seymour K, Kelly SB, et al. Changes in physical activity after bariatric surgery: using objective and self-reported measures. *Surg Obes Relat Dis* 2017;13(3):474-83. doi: 10.1016/j.soard.2016.09.012

28. Raftopoulos I, Bernstein B, O'Hara K, et al. Protein intake compliance of morbidly obese patients undergoing bariatric surgery and its effect on weight loss and biochemical parameters. *Surg Obes Relat Dis* 2011;7(6):733-42. doi: 10.1016/j.soard.2011.07.008

29. Sherf Dagan S, Tovim TB, Keidar A, et al. Inadequate protein intake after laparoscopic sleeve gastrectomy surgery is associated with a greater fat free mass loss. *Surg Obes Relat Dis* 2017;13(1):101-09. doi: 10.1016/j.soard.2016.05.026

30. Moize V, Andreu A, Rodriguez L, et al. Protein intake and lean tissue mass retention following bariatric surgery. *Clinical nutrition* 2013;32(4):550-5. doi: 10.1016/j.clnu.2012.11.007

31. Faria SL, Faria OP, Buffington C, et al. Dietary protein intake and bariatric surgery patients: a review. *Obes Surg* 2011;21(11):1798-805. doi: 10.1007/s11695-011-0441-y

32. Ito MK, Goncalves VSS, Faria S, et al. Effect of Protein Intake on the Protein Status and Lean Mass of Post-Bariatric Surgery Patients: a Systematic Review. *Obes Surg* 2017;27(2):502-12. doi: 10.1007/s11695-016-2453-0

33. Kolotkin RL, Meter K, Williams GR. Quality of life and obesity. *Obes Rev* 2001;2(4):219-29.

34. Munoz DJ, Lal M, Chen EY, et al. Why patients seek bariatric surgery: a qualitative and quantitative analysis of patient motivation. *Obes Surg* 2007;17(11):1487-91.

35. Testa MA, Simonson DC. Assessment of quality-of-life outcomes. *N Engl J Med* 1996;334(13):835-40. doi: 10.1056/NEJM199603283341306

36. Kolotkin RL, Andersen JR. A systematic review of reviews: exploring the relationship between obesity, weight loss and health-related quality of life. *Clinical obesity* 2017 doi: 10.1111/cob.12203

37. Lindekilde N, Gladstone BP, Lubeck M, et al. The impact of bariatric surgery on quality of life: a systematic review and meta-analysis. *Obes Rev* 2015;16(8):639-51. doi: 10.1111/obr.12294

38. Pollock RF, Chilcott J, Muduma G, et al. Laparoscopic adjustable gastric banding vs standard medical management in obese patients with type 2 diabetes: a budget impact analysis in the UK. *Journal of medical economics* 2013;16(2):249-59. doi: 10.3111/13696998.2012.751388

39. Terranova L, Busetto L, Vestri A, et al. Bariatric surgery: cost-effectiveness and budget impact. *Obes Surg* 2012;22(4):646-53. doi: 10.1007/s11695-012-0608-1

40. Ackroyd R, Mouiel J, Chevallier JM, et al. Cost-effectiveness and budget impact of obesity surgery in patients with type-2 diabetes in three European countries. *Obes Surg* 2006;16(11):1488-503. doi: 10.1381/096089206778870067

41. De Luca M, Angrisani L, Himpens J, et al. Indications for Surgery for Obesity and Weight-Related Diseases: Position Statements from the International Federation for the Surgery of Obesity and Metabolic Disorders (IFSO). *Obes Surg* 2016;26(8):1659-96. doi: 10.1007/s11695-016-2271-4

42. Bush K, Kivlahan DR, McDonell MB, et al. The AUDIT alcohol consumption questions (AUDIT-C): an effective brief screening test for problem drinking. Ambulatory Care Quality

For peer review only - http://bmjopen.bmj.com/site/about/guidelines.xhtml

Improvement Project (ACQUIP). Alcohol Use Disorders Identification Test. *Archives of internal medicine* 1998;158(16):1789-95.

43. Lee SY, Gallagher D. Assessment methods in human body composition. *Current opinion in clinical nutrition and metabolic care* 2008;11(5):566-72. doi: 10.1097/MCO.0b013e32830b5f23

44. Faria SL, Faria OP, Cardeal MD, et al. Validation study of multi-frequency bioelectrical impedance with dual-energy X-ray absorptiometry among obese patients. *Obes Surg* 2014;24(9):1476-80. doi: 10.1007/s11695-014-1190-5

45. Migueles JH, Cadenas-Sanchez C, Ekelund U, et al. Accelerometer Data Collection and Processing Criteria to Assess Physical Activity and Other Outcomes: A Systematic Review and Practical Considerations. *Sports medicine* 2017 doi: 10.1007/s40279-017-0716-0

46. A. T. S. Committee on Proficiency Standards for Clinical Pulmonary Function Laboratories. ATS statement: guidelines for the six-minute walk test. *Am J Respir Crit Care Med* 2002;166(1):111-7. doi: 10.1164/ajrccm.166.1.at1102

47. Pataky Z, Armand S, Muller-Pinget S, et al. Effects of obesity on functional capacity. *Obesity (Silver Spring)* 2014;22(1):56-62. doi: 10.1002/oby.20514

48. Sousa-Santos AR, Amaral TF. Differences in handgrip strength protocols to identify sarcopenia and frailty - a systematic review. *BMC geriatrics* 2017;17(1):238. doi: 10.1186/s12877-017-0625-y

49. Yang YJ, Kim MK, Hwang SH, et al. Relative validities of 3-day food records and the food frequency questionnaire. *Nutrition research and practice* 2010;4(2):142-8. doi: 10.4162/nrp.2010.4.2.142

50. Brooks R. EuroQol: the current state of play. *Health policy* 1996;37(1):53-72.

51. Dolan P. Modeling valuations for EuroQol health states. *Medical care* 1997;35(11):1095-108.

52. Kolotkin RL, Crosby RD, Kosloski KD, et al. Development of a brief measure to assess quality of life in obesity. *Obes Res* 2001;9(2):102-11. doi: 10.1038/oby.2001.13

53. Beck AT, Steer RA, Brown GK. Manual for the Beck Depression Inventory-II. San Antonio, TX1996.

54. Beecham J, Knapp M. Costing psychiatric interventions, in G. Thornicroft (ed.) Measuring Mental Health Needs. 2nf edition ed: Gaskell 2001:200-224.

55. Curtis L, Burns A. Unit Costs of Health and Social Care 2015: Personal Social Services Research Unit, University of Kent, Canterbury, 2015.

56. Department of Health. National Schedule of Reference Costs: DOH, London, 2015.

57. British National Formulary. [Available from: https://www.bnf.org/.

58. Barber J, Thompson S. Multiple regression of cost data: use of generalised linear models. *J Health Serv Res Policy* 2004;9(4):197-204. doi: 10.1258/1355819042250249

For peer review only - http://bmjopen.bmj.com/site/about/guidelines.xhtml

Table

Table 1 Participant eligibility criteria for participation in the BARI-LIFESTYLE observational study

**INCLUSION CRITERIA**	
1	Adult aged between 18 to 65 years.
2	Planned to undergo either primary RYGB or SG surgery and fulfilling NICE eligibility criteria for bariatric surgery[1].
3	Able to read and write in English.
4	Willing and able to provide written informed consent.
5	Able to comply with study protocol.
6	Willing and able to wear a Fitbit wrist-based activity tracker device and an ActiGraph device.
**EXCLUSION CRITERIA**	
1	More than 200 kg of body weight due to the limitation of DXA scan.
2	Non-ambulatory.
3	Functional limitation.

For peer review only

For peer review only

For peer review only - http://bmjopen.bmj.com/site/about/guidelines.xhtml

632 **Table 2** Study timeline and investigations

633

	Baseline (T0)	Day of surgery	3-month post-surgery (T1)	6-month post-surgery (T2)	12-month post-surgery (T3)
Sociodemographic data	✓				
Height	✓				
Weight	✓	✓	✓	✓	✓
Blood pressure and heart rate	✓		✓	✓	✓
Dual-energy X-ray absorptiometry scan	✓				✓
Bioelectrical impedance analysis	✓		✓	✓	✓
Laboratory test	✓		✓	✓	✓
Physical activity levels (ActiGraph) and activity diary	✓		✓	✓	✓
Physical function and strength:					
6-minute walk test (6MWT)	✓		✓	✓	✓
Sit-to-stand (STS) test	✓		✓	✓	✓
Handgrip test	✓		✓	✓	✓
Dietary intake (3-day food diary)	✓		✓	✓	✓
Completion of questionnaires:					
EuroQol-5D-3L (EQ-5D-3L)	✓		✓	✓	✓
Impact of weight on Quality of Life-Lite (IWQOL-Lite)	✓		✓	✓	✓
Beck Depression Inventory-II (BDI-II)	✓		✓	✓	✓
Client Service Receipt Inventory (CSRI)	✓		✓	✓	✓
Review of medication	✓		✓	✓	✓
Review of comorbidities	✓		✓	✓	✓

For peer review only

For peer review only - http://bmjopen.bmj.com/site/about/guidelines.xhtml

Figure

Figure 1 Flow diagram of participant enrolment, consent, assessment and associated timeline

For peer review only - http://bmjopen.bmj.com/site/about/guidelines.xhtml

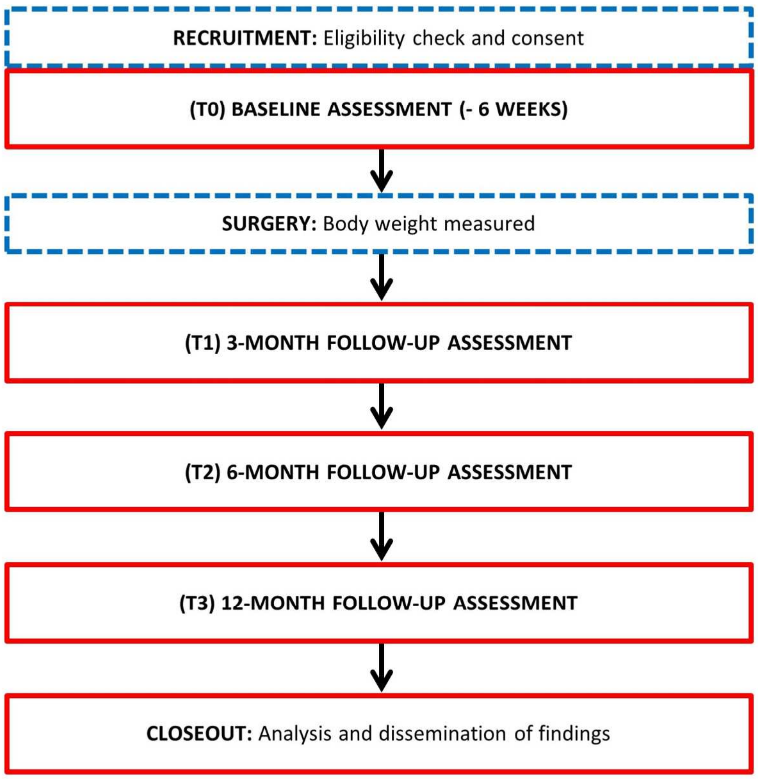

Figure 1 Flow diagram of participant enrolment, consent, assessment and associated timeline

150x150mm (300 x 300 DPI)

SPIRIT — STANDARD PROTOCOL ITEMS: RECOMMENDATIONS FOR INTERVENTIONAL TRIALS

SPIRIT 2013 Checklist: Recommended items to address in a clinical trial protocol and related documents*

Section/item	Item No	Description
**Administrative information**		
Title	1	Descriptive title identifying the study design, population, interventions, and, if applicable, trial acronym<br>**Page 1**
Trial registration	2a	Trial identifier and registry name. If not yet registered, name of intended registry<br>**N/A**
	2b	All items from the World Health Organization Trial Registration Data Set<br>**N/A**
Protocol version	3	Date and version identifier<br>**N/A**
Funding	4	Sources and types of financial, material, and other support<br>**Page 16**
Roles and responsibilities	5a	Names, affiliations, and roles of protocol contributors<br>**Page 1 and page 16**
	5b	Name and contact information for the trial sponsor<br>**N/A**
	5c	Role of study sponsor and funders, if any, in study design; collection, management, analysis, and interpretation of data; writing of the report; and the decision to submit the report for publication, including whether they will have ultimate authority over any of these activities<br>**Page 16**
	5d	Composition, roles, and responsibilities of the coordinating centre, steering committee, endpoint adjudication committee, data management team, and other individuals or groups overseeing the trial, if applicable (see Item 21a for data monitoring committee)<br>**N/A**

For peer review only

Introduction

Background and rationale	6a	Description of research question and justification for undertaking the trial, including summary of relevant studies (published and unpublished) examining benefits and harms for each intervention<br>**Page 4**
	6b	Explanation for choice of comparators<br>**N/A**
Objectives	7	Specific objectives or hypotheses<br>**Page 6**
Trial design	8	Description of trial design including type of trial (eg, parallel group, crossover, factorial, single group), allocation ratio, and framework (eg, superiority, equivalence, noninferiority, exploratory)<br>**Page 7**

Methods: Participants, interventions, and outcomes

Study setting	9	Description of study settings (eg, community clinic, academic hospital) and list of countries where data will be collected. Reference to where list of study sites can be obtained<br>**Page 7**
Eligibility criteria	10	Inclusion and exclusion criteria for participants. If applicable, eligibility criteria for study centres and individuals who will perform the interventions (eg, surgeons, psychotherapists)<br>**Page 21 (Table 1)**
Interventions	11a	Interventions for each group with sufficient detail to allow replication, including how and when they will be administered<br>**N/A**
	11b	Criteria for discontinuing or modifying allocated interventions for a given trial participant (eg, drug dose change in response to harms, participant request, or improving/worsening disease)<br>**N/A**
	11c	Strategies to improve adherence to intervention protocols, and any procedures for monitoring adherence (eg, drug tablet return, laboratory tests)<br>**N/A**
	11d	Relevant concomitant care and interventions that are permitted or prohibited during the trial<br>**N/A**

For peer review only - http://bmjopen.bmj.com/site/about/guidelines.xhtml

Outcomes	12	Primary, secondary, and other outcomes, including the specific measurement variable (eg, systolic blood pressure), analysis metric (eg, change from baseline, final value, time to event), method of aggregation (eg, median, proportion), and time point for each outcome. Explanation of the clinical relevance of chosen efficacy and harm outcomes is strongly recommended<br>**Page 9**
Participant timeline	13	Time schedule of enrolment, interventions (including any run-ins and washouts), assessments, and visits for participants. A schematic diagram is highly recommended (see Figure)<br>**Page 23 (Figure 1) and Page 22 (Table 2)**
Sample size	14	Estimated number of participants needed to achieve study objectives and how it was determined, including clinical and statistical assumptions supporting any sample size calculations<br>**Page 12**
Recruitment	15	Strategies for achieving adequate participant enrolment to reach target sample size<br>**Page 8**

Methods: Assignment of interventions (for controlled trials)

Allocation:

Sequence generation	16a	Method of generating the allocation sequence (eg, computer-generated random numbers), and list of any factors for stratification. To reduce predictability of a random sequence, details of any planned restriction (eg, blocking) should be provided in a separate document that is unavailable to those who enrol participants or assign interventions<br>**N/A**
Allocation concealment mechanism	16b	Mechanism of implementing the allocation sequence (eg, central telephone; sequentially numbered, opaque, sealed envelopes), describing any steps to conceal the sequence until interventions are assigned<br>**N/A**
Implementation	16c	Who will generate the allocation sequence, who will enrol participants, and who will assign participants to interventions<br>**N/A**
Blinding (masking)	17a	Who will be blinded after assignment to interventions (eg, trial participants, care providers, outcome assessors, data analysts), and how<br>**N/A**

	17b	If blinded, circumstances under which unblinding is permissible, and procedure for revealing a participant's allocated intervention during the trial<br>**N/A**

Methods: Data collection, management, and analysis

Data collection methods	18a	Plans for assessment and collection of outcome, baseline, and other trial data, including any related processes to promote data quality (eg, duplicate measurements, training of assessors) and a description of study instruments (eg, questionnaires, laboratory tests) along with their reliability and validity, if known. Reference to where data collection forms can be found, if not in the protocol<br>**Page 9 and Page 22 (Table 2)**
	18b	Plans to promote participant retention and complete follow-up, including list of any outcome data to be collected for participants who discontinue or deviate from intervention protocols<br>**N/A**
Data management	19	Plans for data entry, coding, security, and storage, including any related processes to promote data quality (eg, double data entry; range checks for data values). Reference to where details of data management procedures can be found, if not in the protocol<br>**Page 14**
Statistical methods	20a	Statistical methods for analysing primary and secondary outcomes. Reference to where other details of the statistical analysis plan can be found, if not in the protocol<br>**Page 12**
	20b	Methods for any additional analyses (eg, subgroup and adjusted analyses)<br>**Page 12**
	20c	Definition of analysis population relating to protocol non-adherence (eg, as randomised analysis), and any statistical methods to handle missing data (eg, multiple imputation)<br>**Page 13**

Methods: Monitoring

Data monitoring	21a	Composition of data monitoring committee (DMC); summary of its role and reporting structure; statement of whether it is independent from the sponsor and competing interests; and reference to where further details about its charter can be found, if not in the protocol. Alternatively, an explanation of why a DMC is not needed<br>**N/A**

	21b	Description of any interim analyses and stopping guidelines, including who will have access to these interim results and make the final decision to terminate the trial<br>**N/A**
Harms	22	Plans for collecting, assessing, reporting, and managing solicited and spontaneously reported adverse events and other unintended effects of trial interventions or trial conduct<br>**Page 14**
Auditing	23	Frequency and procedures for auditing trial conduct, if any, and whether the process will be independent from investigators and the sponsor<br>**N/A**
**Ethics and dissemination**		
Research ethics approval	24	Plans for seeking research ethics committee/institutional review board (REC/IRB) approval<br>**Page 13**
Protocol amendments	25	Plans for communicating important protocol modifications (eg, changes to eligibility criteria, outcomes, analyses) to relevant parties (eg, investigators, REC/IRBs, trial participants, trial registries, journals, regulators)<br>**N/A**
Consent or assent	26a	Who will obtain informed consent or assent from potential trial participants or authorised surrogates, and how (see Item 32)<br>**Page 8**
	26b	Additional consent provisions for collection and use of participant data and biological specimens in ancillary studies, if applicable<br>**N/A**
Confidentiality	27	How personal information about potential and enrolled participants will be collected, shared, and maintained in order to protect confidentiality before, during, and after the trial<br>**Page 14**
Declaration of interests	28	Financial and other competing interests for principal investigators for the overall trial and each study site<br>**Page 17**
Access to data	29	Statement of who will have access to the final trial dataset, and disclosure of contractual agreements that limit such access for investigators<br>**Page 14**

For peer review only

Ancillary and post-trial care	30	Provisions, if any, for ancillary and post-trial care, and for compensation to those who suffer harm from trial participation<br>**N/A**
Dissemination policy	31a	Plans for investigators and sponsor to communicate trial results to participants, healthcare professionals, the public, and other relevant groups (eg, via publication, reporting in results databases, or other data sharing arrangements), including any publication restrictions<br>**Page 14**
	31b	Authorship eligibility guidelines and any intended use of professional writers<br>**N/A**
	31c	Plans, if any, for granting public access to the full protocol, participant-level dataset, and statistical code<br>**N/A**
**Appendices**		
Informed consent materials	32	Model consent form and other related documentation given to participants and authorised surrogates<br>**N/A**
Biological specimens	33	Plans for collection, laboratory evaluation, and storage of biological specimens for genetic or molecular analysis in the current trial and for future use in ancillary studies, if applicable<br>**N/A**

*It is strongly recommended that this checklist be read in conjunction with the SPIRIT 2013 Explanation & Elaboration for important clarification on the items. Amendments to the protocol should be tracked and dated. The SPIRIT checklist is copyrighted by the SPIRIT Group under the Creative Commons "Attribution-NonCommercial-NoDerivs 3.0 Unported" license.

For peer review only
